# Synthesis and Biochemical Evaluation of Lid-Open d-Amino Acid Oxidase Inhibitors

**DOI:** 10.3390/molecules24020290

**Published:** 2019-01-14

**Authors:** Bence Szilágyi, Csilla Hargitai, Ádám A. Kelemen, Anita Rácz, György G. Ferenczy, Balázs Volk, György M. Keserű

**Affiliations:** 1 Medicinal Chemistry Research Group, Research Centre for Natural Sciences, Hungarian Academy of Sciences, Magyar tudósok krt. 2, H-1117 Budapest, Hungary; szilagyi.bence@ttk.mta.hu (B.S.); kelemen.adam@ttk.mta.hu (Á.A.K.); ferenczy.gyorgy@ttk.mta.hu (G.G.F.); 2 Directorate of Drug Substance Development, Egis Pharmaceuticals Plc., P.O. Box 100, H-1475 Budapest, Hungary; hargitai.csilla@egis.hu; 3 Plasma Chemistry Research Group, Research Centre for Natural Sciences, Hungarian Academy of Sciences, Magyar tudósok krt. 2, H-1117 Budapest, Hungary; racz.anita@ttk.mta.hu

**Keywords:** d-amino acid oxidase (DAAO), inhibitor, lid-open conformation, Topliss scheme, structure-activity relationship

## Abstract

Most of the known inhibitors of d-amino acid oxidase (DAAO) are small polar molecules recognized by the active site of the enzyme. More recently a new class of DAAO inhibitors has been disclosed that interacts with loop 218−224 at the top of the binding pocket. These compounds have a significantly larger size and more beneficial physicochemical properties than most reported DAAO inhibitors, however, their structure-activity relationship is poorly explored. Here we report the synthesis and evaluation of this type of DAAO inhibitors that open the lid over the active site of DAAO. In order to collect relevant SAR data we varied two distinct parts of the inhibitors. A systematic variation of the pendant aromatic substituents according to the Topliss scheme resulted in DAAO inhibitors with low nanomolar activity. The activity showed low sensitivity to the substituents investigated. The variation of the linker connecting the pendant aromatic moiety and the acidic headgroup revealed that the interactions of the linker with the enzyme were crucial for achieving significant inhibitory activity. Structures and activities were analyzed based on available X-ray structures of the complexes. Our findings might support the design of drug-like DAAO inhibitors with advantageous physicochemical properties and ADME profile.

## 1. Introduction

d-Amino acid oxidase (DAAO) is a flavoprotein that catalyzes the oxidative deamination of d-amino acids. It plays a crucial role in oxidizing d-serine, a co-agonist of the NMDA receptor, whose hypoactivity is thought to be involved in the positive, negative and cognitive symptoms in schizophrenia. Therefore, increased DAAO activity results in lower d-serine level [1,2] and lower NMDA activity. Indeed, it has been shown from *post mortem* brain tissue samples of patients who suffered from schizophrenia that DAAO expression and enzyme activity were elevated compared to healthy controls [3]. These findings suggest that the inhibition of DAAO may result in an increase of brain d-serine level and may have positive effect on the symptoms of schizophrenia [4].

**Figure 1:**
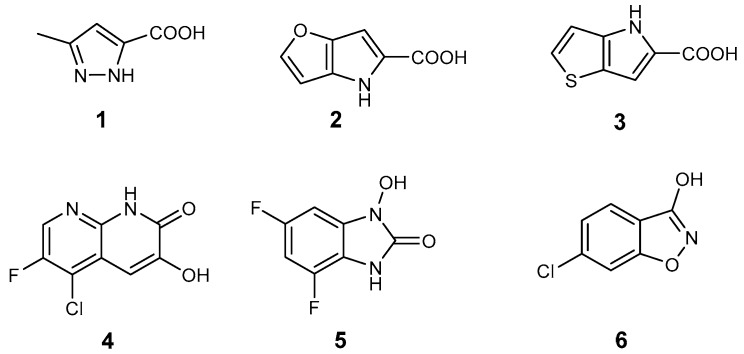
Known active site DAAO inhibitors in the literature.

First generation DAAO inhibitors **1**−**6** [5,6,7,8,9,10] are mostly small polar molecules in accordance with the properties of the enzyme active site (Figure 1). These compounds, however, tend to have suboptimal pharmacokinetic properties. In particular, they are characterized by poor absorption and penetration through the blood-brain barrier.

**Figure 2:**
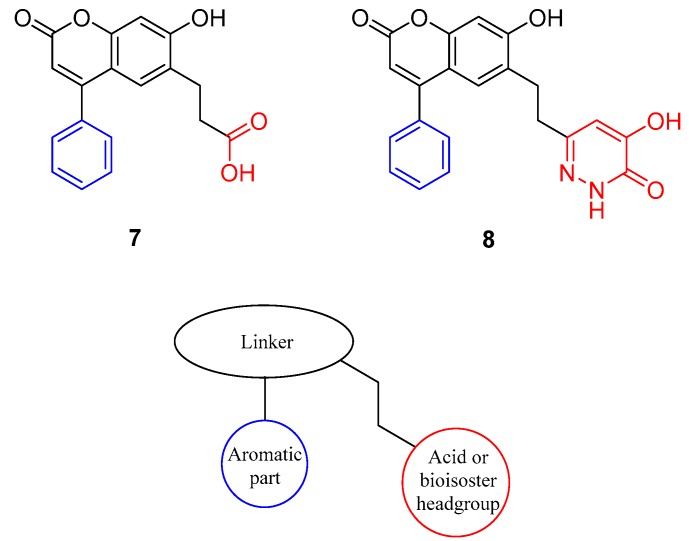
Novel DAAO inhibitors that interact with the flexible loop and the structural moieties of the lid-open type compounds.

In 2014, Terry-Lorenzo et al. [11] reported that during the screening of a computationally prioritized library, a structurally novel compound (**7**) was identified showing competitive d-serine inhibitory properties in the low nanomolar range. An analogue of **7** was synthesized by changing the carboxylic acid group to a bioisosteric hydroxypyridazinone moiety to obtain compound **8** (Figure 2).

Compounds **7** and **8** represent a new generation of DAAO inhibitors because, in contrast to previous active site inhibitors, these compounds also interact with residues at the entrance of the binding pocket. X-Ray structures of the complexes of **7** and **8** with DAAO [11] revealed that the pendant phenyl group interacted with the flexible loop formed by residues 218−224. This loop acts as a lid that covers the entry of the binding pocket when small compounds are bound, and it remains open in the complexes of **7** and **8**. Therefore, the compounds in this series can be used to explore the properties and optimal interactions of the flexible loop (amino acids 218–224). Moreover, the absorption of this compound class is expected to be more favorable than that of small, polar compounds.

Targeting active site lids, if available, is a feasible strategy for enzyme inhibition. Since enzymes with lid-gated active sites operate by an induced fit mechanism [12], here we investigated the impact of different structural elements on lid opening and stabilization. Compounds **7** and **8** can be divided into three structural parts (Figure 2). We can identify an aromatic part which is responsible for maintaining the loop in the open conformation, a linker part which is an aromatic moiety with hydrogen-bond donors and acceptors, and an acid or acid bioisoster headgroup which interacts with Arg283 close to the isoalloxazine ring of flavin adenine dinucleotide (FAD). In this paper we present the design, synthesis and testing of lid-open type analogues with potential DAAO inhibitory activity.

## 2. Results and Discussion

We introduced modifications in the linker and in the pendant aromatic part while we used acidic and acid bioisoster headgroups already described for DAAO inhibitors [11,13,14,15,16]. In the first step, we explored what kind of interactions could be formed between the flexible loop and the aromatic part of the compounds, so we have designed derivatives of compound **8** mono-substituted at the aromatic part. The scheme proposed by Topliss [17] has been applied for the stepwise selection of compounds to be synthesized. This scheme is designed for the systematic optimization of aromatic substituents by identifying the effects of the Hammet constant (σ), the hydrophobic substituent constant (π) and Taft’s steric constant (E_s_) on the activity of the compounds. This stepwise process includes the synthesis and testing of a number of compounds in each step and selection of compounds for the next step based on the observed activities in the previous step. In this way, the variation of a few substituents and the activity measurement of a limited number of compounds allow the exploration of the binding site and the optimization of activity.

The prepared compounds were tested as DAAO inhibitors with the KYNA enzyme inhibitory assay [18]. Although d-serine is a natural substrate of DAAO, its metabolite is not suitable for fluorometric evaluation. However in the applied assay, D-kynurenine can be metabolized by DAAO [19] and the metabolite (KYNA) has a favorable fluorescence property. Thus it can be used for fluorescence measurements [20].

**Figure 3:**
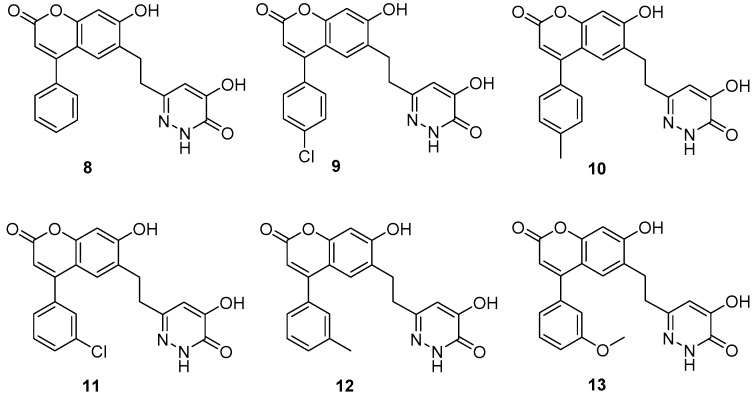
Prepared derivatives of compound **8** monosubstituted at the aromatic part.

**Figure 4:**
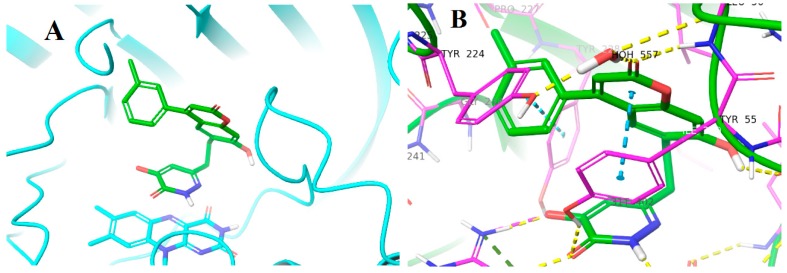
Proposed binding pose of compound **12**. (**A**) Overall structure with protein shown in ribbon diagram, ligand in green tube and the isoalloxazine ring of FAD in cyan tube representation. (**B**) Details of interactions are indicated by dashed lines: yellow hydrogen-bond, cyan aromatic–aromatic. (H-bond criterion: heavy atom distance < 2.8 Å; aromatic-aromatic contact criteria: face-to-face distance < 4.4 Å; face-to edge distance < 5.5 Å).

We started with compounds **8** and **9** (Figure 3). Since they exhibited similar activity (Table 1), we proceeded with the synthesis of **10**. This latter compound showed slightly lower activity, therefore we decided to synthesize **11**. The activity of this 3-Cl substituted derivative (**11**) was lower than 100 nM therefore we synthesized the 3-Me derivative (**12**). Although the X-ray structure of the complex of **8** with DAAO (PDB code: 4QFC [11]) suggested that small substituents in the meta position could be accommodated in the binding pocket, the larger 3-OMe substituent in **13** did not yield further improvement in the activity. DAAO binding and activity of representative compounds have been confirmed in orthogonal differential scanning fluorometry (DSF) and the coupled Horseradish peroxidase (HRP)/Amplex Red assays, respectively (see Supplementary materials). The designed compounds were docked into the protein structure obtained from the complex of **8** by the removal of the ligand. The docking of compounds **9**–**12** resulted in complexes with ligand positions highly similar to that of **8** and suggested that the meta substituent in **11** and **12** points away from the hydroxypyridazinone headgroup (Figure 4).

**Figure 5:**
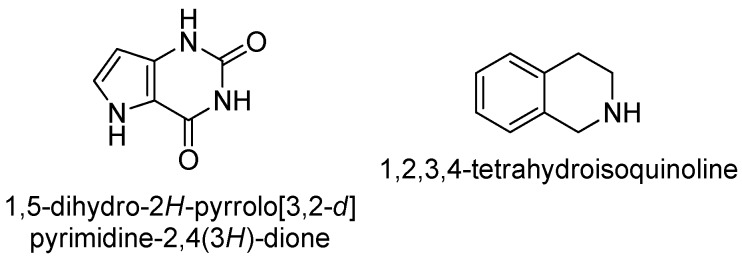
Linkers designed to replace the 7-hydroxy-2*H*-chromen-2-one moiety used in **7** and **8**.

Following the variation of the aromatic substituents, we next focused on the linker region. Starting from the original linker used in compounds **7** and **8,** we selected 1,5-dihydro-2*H*-pyrrolo[3,2-*d*]pyrimidine-2,4(3*H*)-dione and 1,2,3,4-tetrahydroisoquinoline skeletons that might resemble in shape and possible substitution pattern (Figure 5).

**Figure 6:**
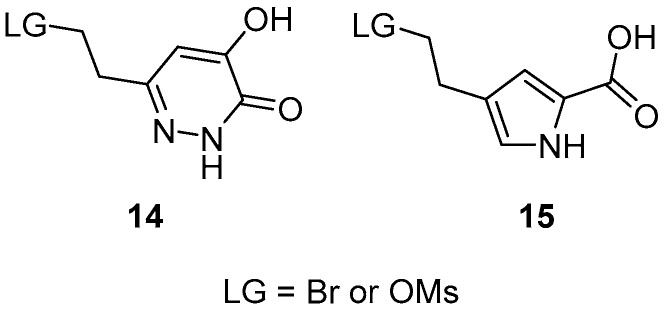
Selected headgroups for the synthesis of lid-open DAAO inhibitors.

As regards the headgroup, the best option would have been the hydroxypyridazinone moiety used in the original compound (**8**), but we failed to produce derivative **14** (Figure 6) that would have been suitable for the coupling reaction. Therefore, we decided to attach a known acidic headgroup of the pyrrole-2-carboxylic acid by using derivative **15**.

**Figure 7:**
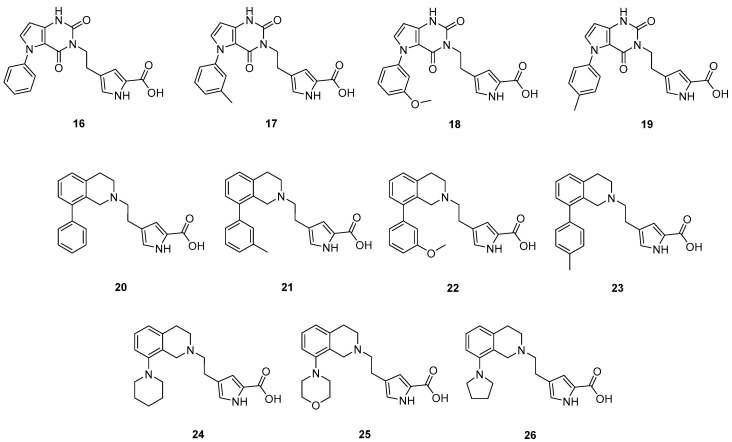
Prepared compounds with new linker part.

The prepared new compounds **16**–**26** (Figure 7) were tested in the KYNA enzyme inhibitory assay, and they did not show any inhibitory activity at 20 μM concentration (see Supplementary materials). Comparing these compounds with DAAO inhibitors **8**–**13** and with pyrrole-2-carboxylic acid containing inhibitors known from the literature [16,21] strongly suggests that it is the linker part of molecules **16**–**26** that prevents DAAO activity. The replacement of the 7-hydroxy-2*H*-chromen-2-one linker of compounds **7**–**13** changes the interactions of the linker, in addition it modifies the exit vectors of both the acidic headgroup and the pendant aromatic moiety in compounds **16**–**19**. An analysis of the X-ray structure of complexes of **7** and **8** (PDB codes: 4QFD [11] and 4QFC [11]) suggests that the loss of linker planarity in compounds **20**–**26** is likely to contribute to the loss of activity as the planar linker of **7** and **8** beneficially interacts with Tyr55, Leu215 and Tyr224, while these stacking and hydrophobic interactions may not be optimally formed with the nonplanar tetrahydroisoquinoline group. Furthermore, the OH group of the 7-hydroxy-2*H*-chromen-2-one linker in **7** and **8** is able to form H-bond with the Gln53 backbone carbonyl and the carbonyl group of the linker forms a water-mediated H-bond to Tyr224. These H-bonds are clearly missing in potential complexes of **20**–**26**. While the presence of H-bond to the Gln53 backbone is highly possible in DAAO complexes of **16**–**19**, no H-bond acceptor to the water-mediated interaction with Tyr224 can be assumed. Perhaps more importantly, the different orientation of the pendant aromatic group in **16**–**19** compared to compounds **9**–**13** might be responsible for the decreased activity. This group in derivatives **9**–**13** points toward the flexible loop formed by residues 218−224 and may not be able to adopt a position beneficially interacting with the loop. These observations suggest that the linker contributes significantly to DAAO activity both by its interactions with the enzyme and by properly orienting the pendant aromatic moiety.

## 3. Materials and Methods

### 3.1. General Information

Melting points were determined on an OptiMelt SRS instrument (Stanford Research Systems, Sunnyvale, CA, USA) and are uncorrected. NMR measurements were performed on a System 500 NMR spectrometer (Varian, Palo Alto, CA, USA) or a Varian System 300 spectrometer. ^1^H- and ^13^C-NMR spectra were measured at room temperature (25 °C) in an appropriate solvent. ^1^H and ^13^C chemical shifts are expressed in parts per million (δ) referenced to TMS or residual solvent signals. Reactions were monitored with silica gel 60 F_254_ TLC plates (Merck, Darmstadt, Germany). All chemicals and solvents were used as purchased. HPLC-MS measurements were performed using a LC-MS-2020 device (Shimadzu, Kyoto, Japan) equipped with a Reprospher 100 C18 (5 µm, 100 × 3 mm) column and positive-negative double ion source (DUIS±) with a quadrupole mass spectrometer in a range of 50–1000 *m*/*z*. Samples were eluted with gradient elution using eluent A (0.1% formic acid in water) and eluent B (0.1% formic acid in acetonitrile). Flow rate was set to 1.5 mL/min. The initial condition was 0% B eluent, followed by a linear gradient to 100% B eluent by 2 min, from 2 to 3.75 min 100% B eluent was retained, and from 3.75 to 4.5 min back to initial condition and retained to 5 min. The column temperature was kept at 30 °C and the injection volume was 1 µL. High resolution mass spectrometric measurements were performed using a Q-TOF Premier mass spectrometer (Waters, Milford, MA, USA) in positive electrospray ionization mode. Compound **8** was prepared following the procedure reported in ref. [11]. Preparation of compounds **9**–**13** and **16**–**26** is described in the Supplementary materials.

### 3.2. KYNA Enzyme Inhibition Assay

d-2-Amino-4-(2-aminophenyl)-4-oxobutanoic acid (D-KYN) was used to measure d-amino acid oxidase activity based on the protocol described in ref. [18,20]. Human DAAO (purchased from TargetEx Ltd. (Dunakeszi, Hungary)) was used for the measurements. The buffer contained 20 mM TRIS-HCl and 100 mM NaCl (pH = 8). Flavin adenine dinucleotide (FAD—obtained from Sigma (St. Louis, MO, USA)) in 5 µM concentration was also included in the assay. Compounds were dissolved originally in DMSO and the measured samples were diluted with the buffer solution (the final DMSO concentration was always below 5%). The mixed solution was incubated at 37 °C for 1 h. After the enzymatic reaction, ZnCl_2_ dissolved in H_2_O was added and vortex-mixed. Single point measurements were performed at 20 µm inhibitor concentration. For the IC_50_ measurements, the inhibitors were used at 5 nM, 50 nM, 500 nM, 2.5 µM, 5 µM, 10 µM, 50 µM. Measurements were carried out on a Citation3 cell imaging multi-mode microplate reader (BioTek Instruments Inc., Winooski, VT, USA) with 364 well plates. The applied wavelengths were 340 nm and 396 nm.

### 3.3. Molecular Modeling

Compounds were docked into the DAAO structure (PDB: 4QFC). Protein and ligand preparations were performed with Schrödinger’s tools [22] with standard settings and Glide [22] was used for docking and scoring.

## 4. Conclusions

In the current investigation, we synthesized and tested compounds derived from DAAO inhibitors that, in contrast to most other reported inhibitors, interact with loop 218−224 at the top of the ligand binding pocket. This loop must be in the open position to allow for substrate binding and typically closes when the ligand is within the pocket. However, large ligands force the loop to leave the pocket partially open after ligand binding and this opens up new opportunities in the development of DAAO inhibitors. We varied two moieties of extended DAAO inhibitors, namely the pendant aromatic group and the linker that connects the former with the acid or acid mimetic headgroup. The compounds produced are of synthetic and medicinal chemistry value, as they explore structure-activity relationship of lid-open DAAO inhibitors. Moreover, a systematic variation of the aromatic substituents according to the scheme proposed by Topliss resulted in DAAO inhibitors in the 2−3 digit nanomolar affinity range. The activity showed low sensitivity to the substituents investigated. The variation of the linker part revealed that the interactions of the linker with the enzyme were crucial for achieving significant inhibitory activity. The analysis of the structures and their activities suggests that a planar linker with H-bond forming ability at suitable positions is indispensable for DAAO activity. These results may find use in DAAO inhibitor design since these types of compounds broaden the operational space to develop inhibitors with advantageous physicochemical properties and ADME profile.

## Figures and Tables

**Table 1:** Enzyme assay test results of compounds **8**–**13**.

Compound	IC_50_ [nM] *
**8**	112(17)
**9**	119(24)
**10**	474(12)
**11**	69(18)
**12**	52(9)
**13**	73(11)

* Standard deviations are shown in parenthesis.

## Supplementary Materials

The following are available online. Scheme S1. Synthesis of the derivatives of 8 substituted on the benzene ring of the aromatic part, Scheme S2. Preparation of compounds **16**–**26**, Scheme S3. Preparation of linkers containing a substituted aromatic moiety, Scheme S4. Synthesis of the headgroup, results from differential scanning fluorimetry (DSF) measurements, results from the coupled HRP/Amplex Red assay for compound, limit dose (20 μM) screening results obtained for compounds **8**–**13** and **16**–**26**.
