# Supplementary material for: Synthesis and Biochemical Evaluation of Lid-Open d-Amino Acid Oxidase Inhibitors
Source: Molecules. 2019 Jan 14;24(2):290. doi: 10.3390/molecules24020290 (PMC6358909; doi:10.3390/molecules24020290)
Supplement: Supplementary file 1 [file molecules-24-00290-s001.pdf]

Supplementary Material

Synthesis and Biochemical Evaluation of Lid-Open D-Amino Acid Oxidase Inhibitors

Bence Szilágyi [1], Csilla Hargitai [2], Ádám A. Kelemen [1], Anita Rácz [3], György G. Ferenczy [1], Balázs Volk [2,*] and György M. Keserű [1,*]

[1] Medicinal Chemistry Research Group, Research Centre for Natural Sciences, Hungarian Academy of Sciences, Magyar tudósok krt. 2, H-1117 Budapest, Hungary; szilagyi.bence@ttk.mta.hu (B.S.); kelemen.adam@ttk.mta.hu (Á.A.K.); ferenczy.gyorgy@ttk.mta.hu (G.G.F.)

[2] Directorate of Drug Substance Development, Egis Pharmaceuticals Plc., P.O. Box 100, H-1475 Budapest, Hungary; hargitai.csilla@egis.hu (C.H.)

[3] Plasma Chemistry Research Group, Research Centre for Natural Sciences, Hungarian Academy of Sciences, Magyar tudósok krt. 2, H-1117 Budapest, Hungary; racz.anita@ttk.mta.hu (A.R.)

* Correspondence: volk.balazs@egis.hu (B.V.); keseru.gyorgy@ttk.mta.hu (G.M.K.); Tel.: +36-1-803-5874 (B.V.); Tel.: +36-1-382-6821 (G.M.K.)

Contents

Synthesis of compounds **9–13** 1
Synthesis of compounds **16–26** 3
Results from differential scanning fluorimetry (DSF) measurements 4
Result from the coupled HRP/Amplex Red assay for compound **13** 5
Limit dose (20 μM) screening results obtained for compounds **8–13** and **16–26** 6
Experimental 6
References 17

Synthesis of compounds 9–13

The preparation of the target compounds was realized by coupling three components (Scheme S1). The precursor of the head group **28** [1] was prepared from 3,4,6-trichloropyridazine (**27**) in a nucleophilic substitution reaction carried out with benzyl alcohol. Derivatives **31a**–**f** [2] carrying the corresponding substituent of the aromatic moiety were prepared from the appropriate acetophenone derivatives **29a**–**f** using diethyl carbonate (**30**). The synthesis of the linker part was started from resorcinol (**32**). First, benzyl protecting groups were applied to the molecule **33** [3] and then iodine was introduced to give **34** [4]. Following a Sonogashira reaction with trimethylsilylacetylene leading to **35**, compound **36** [5] as the third building block was obtained by removing the trimethylsilyl group of **35**. This was followed by linking the components. First, compounds **28** and **36** were coupled and the benzyl protecting groups of derivative **37** were removed. The resulting **38** [6] was coupled with the appropriately substituted benzoylacetic acid ethyl ester derivatives **31a**–**f** to give the expected products **8–13**.

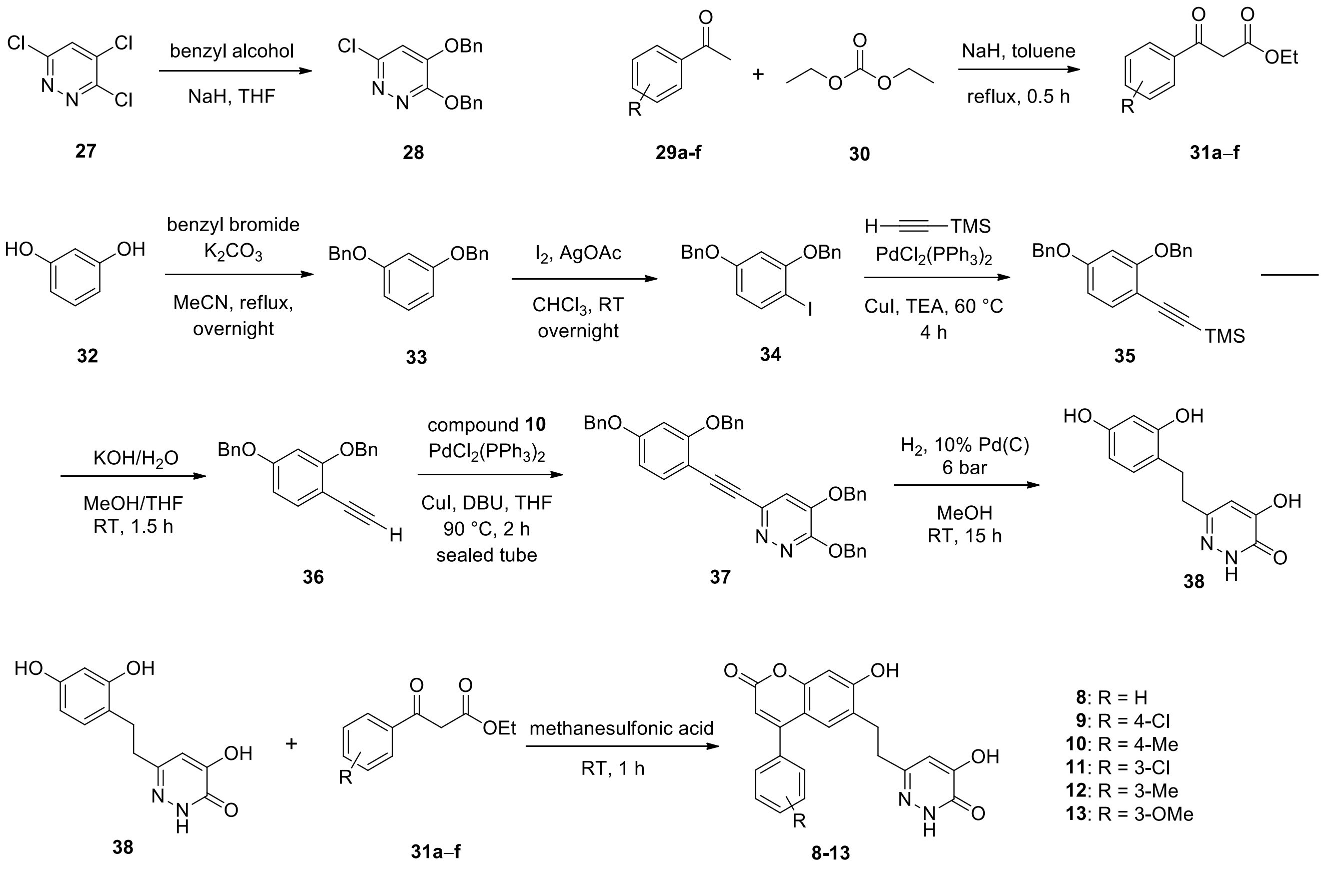

Scheme S1. Synthesis of the derivatives of 8 substituted on the benzene ring of the aromatic part.

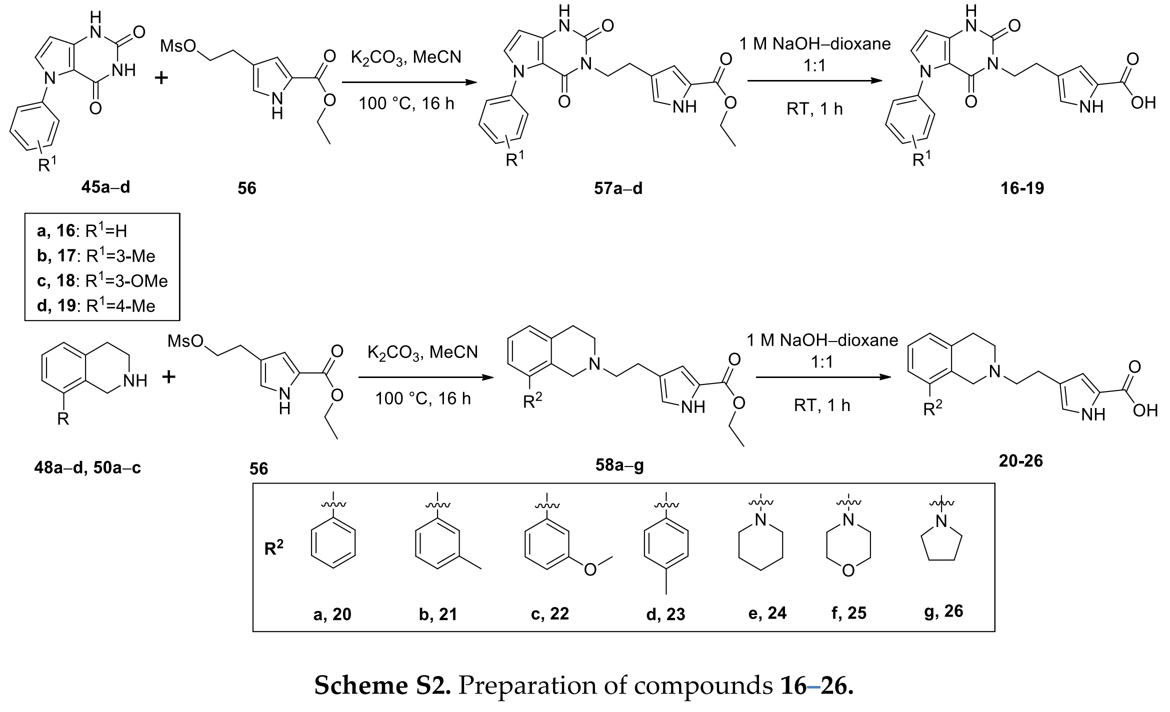

Scheme S2. Preparation of compounds **16–26.**

Synthesis of compounds 16–26

The preparation of the target compounds was realized by coupling two components: the appropriate linker **45a–d**, **48a–d** and **50a–c** equipped with the substituted aryl group and the head group **56** (see Scheme S2). The synthesis of the aryl substituted linkers **45a–d**, **48a–d** and **50a–c** is shown in Scheme S3. The head group **56** was prepared as shown in Scheme S4.

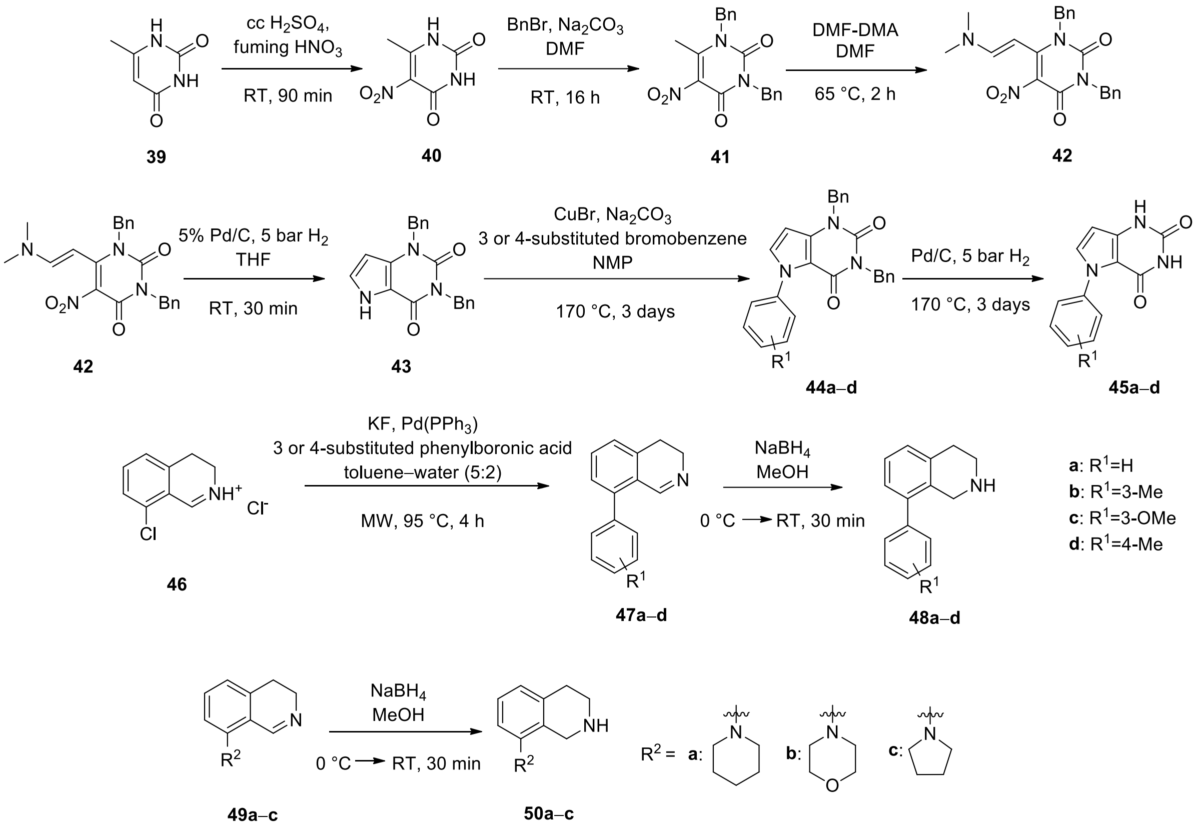

Scheme S3. Preparation of linkers containing a substituted aromatic moiety.

For the preparation of 1,5-dihydro-2*H*-pyrrolo[3,2-*d*]pyrimidine-2,4(3*H*)–dione derivatives **45a**–**d**, 6-methyluracil (**39**) was used as the starting material (Scheme). In the first step, it was nitrated to give **40** [7] followed by the introduction of benzyl protecting groups to give compound **41**. Then, **42** was prepared with dimethylformamide dimethyl acetal (DMF–DMA). The cyclization of the second ring occurred after the nitro group was reduced. For derivatization, the resulting molecule **43** was reacted with bromobenzene derivatives substituted at position 3 or 4 in the presence of a copper catalyst. Following the coupling reactions, the benzyl protecting groups were removed to provide the linker derivatives with the desired aromatic moiety **45a**–**d**.

In case of the 1,2,3,4-tetrahydroisoquinoline compounds **48a**–**d**, the synthesis was carried out starting from 8-chloro-3,4-dihydroisoquinoline hydrochloride (**46**, Scheme) [8]. The derivatization was carried out by palladium(0) catalyzed coupling reaction using the corresponding phenylboronic acids, to give compounds **47** followed by the reduction of the 3,4-dihydroisoquinoline ring to 1,2,3,4-

tetrahydroisoquinoline with sodium borohydride. The synthesis of the headgroup **56** has been completed according to Scheme S4.

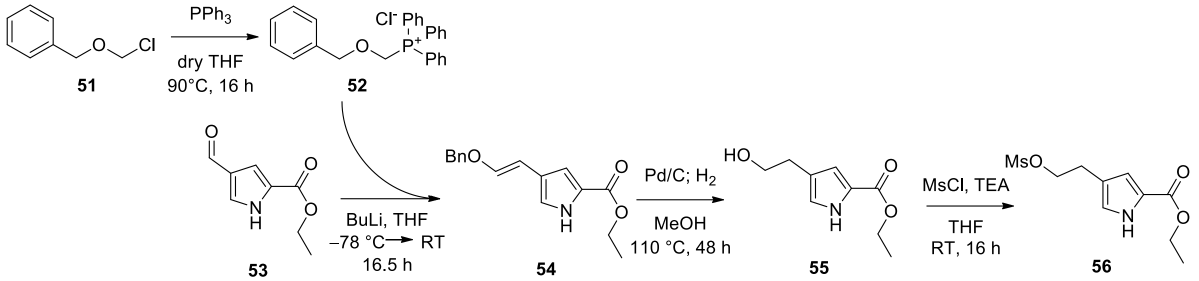

Scheme S4. Synthesis of the headgroup

The formation of the dimethylene spacer was accomplished by a Wittig reaction, however, for this reaction, the corresponding phosphine salt had to be prepared. First, chloromethyl-benzyl ether (**51**) was reacted with triphenylphosphine (Scheme). The thus-formed phosphonium salt **52** was converted under basic conditions to the corresponding phosphorane that was then reacted with compound **53**. After the isolation of the expected derivative **54**, the benzyl group was removed, and the double bond of the side chain was saturated in one step by catalytic hydrogenation. The resulting compound **55** was reacted with methanesulfonic acid chloride to form **56** equipped by the appropriate leaving group for the alkylation reaction. Finally, the headgroup was coupled to the linker derivatives using inorganic bases in acetonitrile at 100 °C (**Error! Reference source not found.**). In the case of pyrrolopyrimidine linkers **45a–d**, the different basicity of the amide nitrogen atoms was used to carry out the reaction regioselectively. Here, test reactions were carried out using various inorganic bases ($NaHCO_3$, Na_2CO_3 and K_2CO_3). Potassium carbonate was the first base the use of which led to the formation of the desired products **57a−d**, while the other isomer was not formed in any case. After the coupling, carboxylic acid esters were hydrolyzed in dioxane−water (1:1) to give the target compounds **16–19**. Reactions starting from tetrahydroisoquinolines **48a−d** and **50a−c** were accomplished in an analogous manner, via esters **58a−g** that were finally hydrolized to the target compounds **20–26**.

Results from differential scanning fluorimetry (DSF) measurements

**Compound**	**Structure**	***DSF measurement***	
		**T_m (°C)**	**~ΔT_m (°C)**
hDAO (no inhibitor)	---	59.4	--
Compound **8**	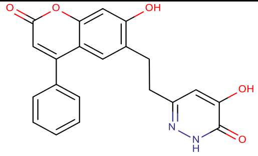	74.7	15.3
Compound **13**	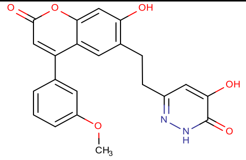	72.0	12.6

D3 (reference)	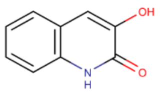	75.1	15.7

Result from the coupled HRP/Amplex Red assay for compound 13

Compound **13** was evaluated in both the KYNA and the coupled HRP/Amplex red assays. Assay conditions are summarized in the table below.

	HRP/Amplex assay	KYNA assay
Substrate	D-Ala	D-Kyn
hDAAO original concentration	1 μg / 1500 μL buffer	1 μg / 400 μL buffer
hDAAO assay concentration	0.25 μg / 1500 μL buffer	0.25 μg / 400 μL buffer
FAD conc. in assay	5 μM	5 μM
Ligand conc. in assay	20 μM	20 μM
Buffer	Tris	Tris
Buffer pH	8	8
Incubation period	No	Yes (1 hour, 37 °C)
Type of the measurement	Kinetic (45 min)	single point detection
Detection	fluorescent	fluorescent
Excitation	544	340
Emission	590	396
Assay interference	Yes	No
Ligand autofluorescence	No	No

Dose response curve obtained for compound **13** using the HRP/Amplex red assay

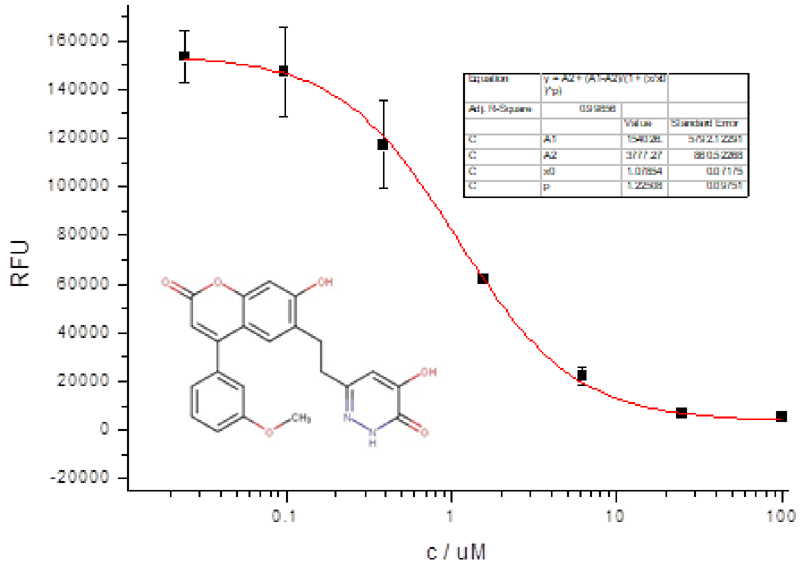

Limit dose (20 µM) screening results obtained for compounds 8–13 and 16–26

Inhibition percentages with standard deviations are plotted in the figure below.

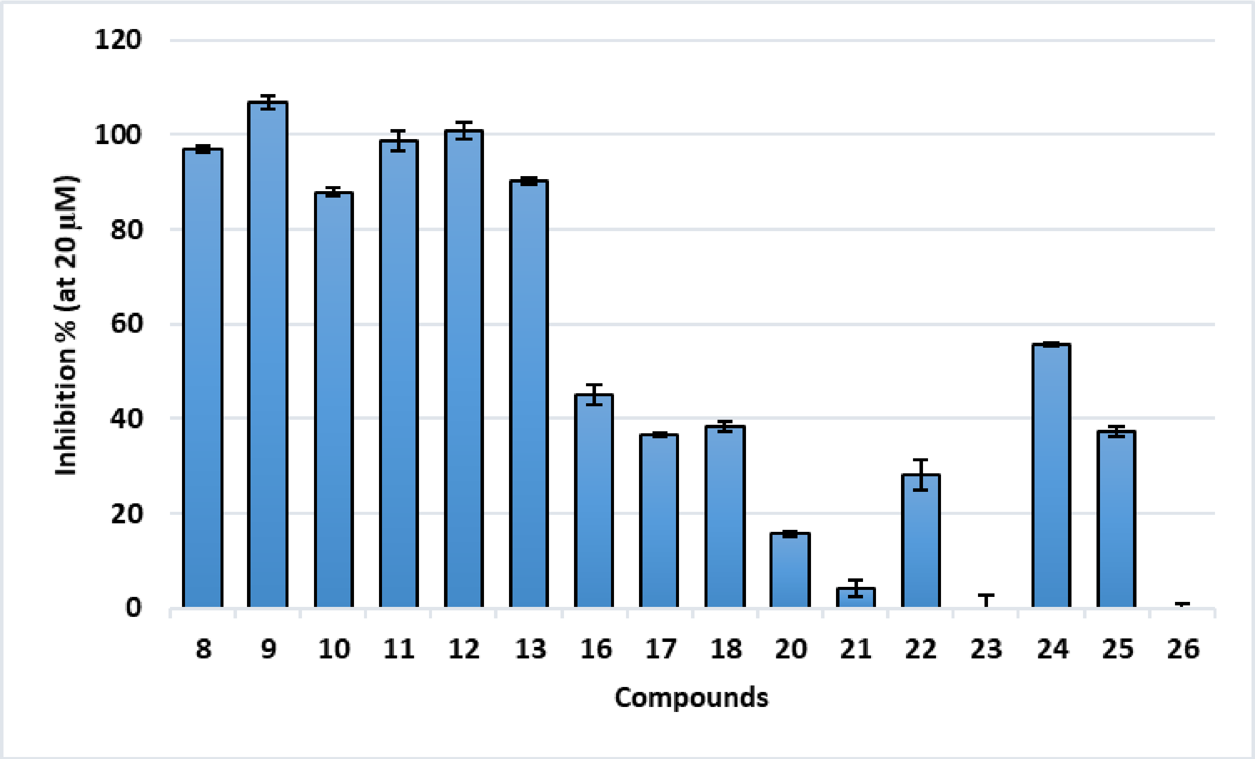

Experimental

General Information

Melting points were determined on an OptiMelt SRS (Sunnyvale, CA, USA) and are uncorrected. NMR measurements were performed on System 500 NMR spectrometer (Varian, Palo Alto, CA, USA) or a Varian System 300 NMR spectrometer, ^{1}H and ^{13}C-NMR spectra were measured at room temperature (25 °C) in an appropriate solvent. ^{1}H and ^{13}C chemical shifts are expressed in parts per million (δ) referenced to TMS or residual solvent signals. Reactions were monitored with silica gel 60 F_{254} TLC plates (Merck, Darmstadt, Germany). All chemicals and solvents were used as purchased. HPLC-MS measurements were performed using a LC-MS-2020 device (Shimadzu, Kyoto, Japan) equipped with a Reprospher 100 C18 (5 µm, 100 × 3mm) column and positive-negative double ion source (DUIS±) with a quadrupole mass spectrometer in a range of 50-1000 *m/z*. Sample was eluted with gradient elution using eluent A (0.1% formic acid in water) and eluent B (0.1% formic acid in acetonitrile). Flow rate was set to 1.5 mL/min. The initial condition was 0% B eluent, followed by a linear gradient to 100% B eluent by 2 min, from 2 to 3.75 min 100% B eluent was retained, and from 3.75 to 4.5 min back to initial condition and retained to 5 min. The column temperature was kept at 30 °C and the injection volume was 1 µl. High resolution mass spectrometric measurements were performed using a Q-TOF Premier mass spectrometer (Waters, Milford, MA, USA) in positive electrospray ionization mode.

Chemistry

6-{2-[4-(4-Chlorophenyl)-7-hydroxy-2-oxo-2H-chromen-6-yl]ethyl}-4-hydroxypyridazin-3(2H)-one (**9**). To the solution of 6-[2-(2,4-dihydroxyphenyl)ethyl]-4-hydroxypyridazin-3(2*H*)-one (**38**, 100 mg, 0.40 mmol) in methanesulfonic acid (5 mL) was added ethyl 3-(4-chlorophenyl)-3-oxopropanoate (**31a**, 91 mg, 0.40 mmol). The reaction mixture was stirred at RT for 1 h, then it was quenched with distilled water (10 mL), and the precipitate was filtered and washed with isopropyl alcohol (5 mL) and purified by preparative HPLC (water–acetonitrile 10% to 100% acetonitrile) to give compound **9** (9.0 mg, 0.022 mmol, 6% yield). ^{1}H-NMR (500 MHz, DMSO-d_6) δ 10.76 (1H, s), 7.58 (d, *J* = 8.3 Hz, 2H), 7.36 (d, *J* = 7.4 Hz, 2H), 6.90 (1H, s), 6.82 (1H, s), 6.52 (1H, s), 6.10 (1H, s), 2.78 (t, *J* = 6.9 Hz, 2H), 2.64 (t, *J* = 6.9 Hz, 2H). HRMS (ESI$^+$) *m/z* $[M+H]^+$ calcd. for $C_{21}H_{16}N_2O_5Cl$: 411.0748, found: 411.0748.

4-Hydroxy-6-{2-[7-hydroxy-4-(4-methylphenyl)-2-oxo-2H-chromen-6-yl]ethyl}pyridazin-3(2H)-one (**10**). To the solution of 6-[2-(2,4-dihydroxyphenyl)ethyl]-4-hydroxypyridazin-3(2*H*)-one (**38**, 100 mg, 0.40 mmol) in methanesulfonic acid (5 mL) was added ethyl 3-(4-methylphenyl)-3-oxopropanoate (**31b**, 82 mg, 0.40 mmol). The reaction mixture was stirred at RT for 1 h, then it was quenched with distilled water (10 mL), and the precipitate was filtered and washed with isopropyl alcohol (5 mL) and purified by preparative HPLC (water–acetonitrile 10% to 100% acetonitrile) to give the title compound **10** (7.0 mg, 0.018 mmol, 5% yield). ^{1}H-NMR (500 MHz, DMSO-d_6) δ 10.70 (s, 1H), 7.33 (d, *J* = 7.3 Hz, 2H), 7.22 (d, *J* = 7.8 Hz, 2H), 6.95 (s, 1H), 6.81 (s, 1H), 6.50 (s, 1H), 6.04 (s, 1H). HRMS (ESI$^+$) *m/z* $[M+H]^+$ calcd. for $C_{22}H_{19}N_2O_5$: 391.1294, found: 391.1293.

6-{2-[4-(3-Chlorophenyl)-7-hydroxy-2-oxo-2H-chromen-6-yl]ethyl}-4-hydroxypyridazin-3(2H)-one (**11**). To the solution of 6-[2-(2,4-dihydroxyphenyl)ethyl]-4-hydroxypyridazin-3(2*H*)-one (**38**, 100 mg, 0.40 mmol) in methanesulfonic acid (5 mL) was added ethyl 3-(3-chlorophenyl)-3-oxopropanoate (**31c**, 91 mg, 0.40 mmol). The reaction mixture was stirred at RT for 1 h, then it was quenched with distilled water (10 mL). The

precipitate was filtered and washed with isopropyl alcohol (5 mL) and purified by preparative HPLC (water–acetonitrile 10% to 100% acetonitrile) to give compound **11** (8.0 mg, 0.019 mmol, 12% yield). ^{1}H-NMR (500 MHz, DMSO-d_6) δ 10.75 (s, 1H), 7.59–7.52 (m, 3H), 7.24 (d, *J* = 7.4 Hz, 1H), 6.92 (s, 1H), 6.82 (s, 1H), 6.48 (s, 1H), 6.14 (s, 1H), 3.46–3.42 (m, 2H), 3.41–3.38 (m, 2H). HRMS (ESI$^+$) *m/z* [M+H]$^+$ calcd. for $C_{21}H_{16}N_2O_5Cl$: 411.0748, found: 411.0747.

4-Hydroxy-6-{2-[7-hydroxy-4-(3-methylphenyl)-2-oxo-2H-chromen-6-yl]ethyl}pyridazin-3(2H)-one (**12**). To a solution of 6-[2-(2,4-dihydroxyphenyl)ethyl]-4-hydroxypyridazin-3(2*H*)-one (**38**, 100 mg, 0.40 mmol) in methanesulfonic acid (5 mL) was added ethyl 3-(3-methylphenyl)-3-oxopropanoate (**31d**, 82 mg, 0.40 mmol). The reaction mixture was stirred at RT for 1 h, then it was quenched with distilled water (10 mL). The precipitate was filtered and washed with isopropyl alcohol (5 mL) and purified by preparative HPLC (water–acetonitrile 10% to 100% acetonitrile) to give compound **12** (6.0 mg, 0.015 mmol, 4% yield). ^{1}H-NMR (500 MHz, DMSO-d_6) δ 10.70 (s, 1H), 7.39 (t, J = 7.6 Hz, 1H), 7.32 (m, 1H), 7.22 (s, 1H), 7.07 (d, J = 7.6 Hz, 1H), 6.96 (s, 1H), 6.81 (s, 1H), 6.46 (s, 1H), 6.05 (s, 1H). HRMS (ESI$^+$) *m/z* [M+H]$^+$ calcd. for $C_{22}H_{19}N_2O_5$: 391.1294, found: 391.1293.

4-Hydroxy-6-{2-[7-hydroxy-4-(3-methoxyphenyl)-2-oxo-2H-chromen-6-yl]ethyl}pyridazin-3(2H)-one (**13**). To the solution of 6-[2-(2,4-dihydroxyphenyl)ethyl]-4-hydroxypyridazin-3(2*H*)-one (**38**, 100 mg, 0.40 mmol) in methanesulfonic acid (5 mL) was added ethyl 3-(3-methoxyphenyl)-3-oxopropanoate (**31e**, 89 mg, 0.40 mmol). The reaction mixture was stirred at RT for 1 h, then it was quenched by distilled water (10 mL). The precipitate was filtered and washed with isopropyl alcohol (5 mL) and purified by preparative HPLC (water−acetonitrile 10% to 100% acetonitrile) to give compound **13** (14.0 mg, 0.035 mmol, 9%). ^{1}H-NMR (500 MHz, DMSO-d_6) δ 10.76 (1H, s), 7.44 (t, *J* = 8.0 Hz, 1H), 7.09 (m, 1H), 7.04 (m, 2H), 6.85 (m, 2H), 6.51 (s, 1H), 6.13 (s, 1H), 3.81 (s, 3H), 2.79 (t, *J* = 7.1 Hz, 2H), 2.64 (t, *J* = 7.1 Hz, 2H). ^{13}C-NMR (500 MHz, DMSO-d_6) δ 160.60, 159.95, 159.81, 158.22, 155.62, 154.34, 136.92, 130.22, 128.05, 125.29, 121.96, 120.86, 115.71, 114.02, 110.72, 110.56, 109.11, 102.63, 55.31, 34.36, 28.69. HRMS (ESI$^+$) *m/z* [M+H]$^+$ calcd. for $C_{22}H_{19}N_2O_6$: 407.1243, found: 407.1243.

1,3-Dibenzyl-1H-pyrrolo[3,2-d]pyrimidine-2,4(3H,5H)-dione (**43**).

To the solution of 6-methyl-5-nitropyrimidine-2,4(1*H*,3*H*)-dione (**40**, 5.00 g, 29.2 mmol) in DMF (190 mL), Na_2CO_3 (7.75 g, 73.1 mmol) and benzyl bromide (8.67 mL, 73.0 mmol) were added. The reaction mixture was stirred at RT for 16 h, then water (270 mL) was added. The solution was washed with ethyl acetate (3 × 250 mL). The combined organic phase was dried over $MgSO_4$, filtered and evaporated. The combined organic phase was dried over $MgSO_4$, filtered and evaporated to give 1,3-dibenzyl-6-methyl-5-nitropyrimidine-2,4(1*H*,3*H*)-dione (**41**, 8.03 g, 22.8 mmol, 78% yield) as pale yellow crystals. Mp 134−136 °C. LC-MS [M+H]$^+$: 352 *m/z*. ^{1}H-NMR (500 MHz, $CDCl_3$) δ 7.51 (dd, *J* = 7.7, 1.3 Hz, 2H), 7.40–7.27 (m, 6H), 7.15 (d, *J* = 7.1 Hz, 2H), 5.20 (s, 4H), 2.31 (s, 3H). ^{13}C-NMR (500 MHz, $CDCl_3$) δ 154.85, 150.64, 149.55, 135.71, 134.62, 129.53, 129.51, 128.73, 128.58, 128.31, 126.35, 126.34, 49.09, 45.92, 16.09.

To the solution of 1,3-dibenzyl-6-methyl-5-nitropyrimidine-2,4(1*H*,3*H*)-dione (**41**, 10.00 g, 28.5 mmol) in DMF (20 mL), DMF–DMA (7.4 mL, 72.7 mmol) was added and stirred for 2 h at 65 °C. The reaction mixture was cooled to RT, methanol (5 mL) was added, then diethyl ether (100 mL) was added dropwise. The orange solid was filtered, washed with diethyl ether (20 mL), then dried to give 1,3-dibenzyl-6-[(*E*)-2-(dimethylamino)ethenyl]-5-nitropyrimidine-2,4(1*H*,3*H*)-dione (**42**, 9.10 g, 22.4 mmol, 79% yield). Mp 155–157 °C. LC-MS [M+H]$^+$: 407 *m/z*. ^{1}H-NMR (500 MHz, $CDCl_3$) δ 7.53 (d, *J* = 6.9 Hz, 2H), 7.38–7.27 (m, 6H), 7.20 (d, *J* = 7.2 Hz, 2H), 6.87 (d, *J* = 12.8 Hz, 1H), 5.17 (d, *J* = 7.2 Hz, 4H), 4.43 (d, *J* = 12.9 Hz, 1H), 2.83 (s, 6H).

^{13}C-NMR (500 MHz, $CDCl_3$) δ 155.65, 151.29, 150.50, 150.07, 136.62, 136.18, 129.41, 129.23, 128.54, 127.96, 127.86, 126.30, 81.21, 49.79, 45.36.

To the solution of 1,3-dibenzyl-6-[(*E*)-2-(dimethylamino)ethenyl]-5-nitropyrimidine-2,4(1*H*,3*H*)-dione (**42**, 1.00 g, 2.46 mmol) in THF (40 mL), Pd on carbon (100 mg) was added, and hydrogenated under 5 bar H_2 pressure for 30 min. The suspension was filtered through Celite and washed with THF (2 × 10 mL). The crued product was purified by flash column chromatography (hexane–ethyl acetate, from 1:0 to 7:3) to give compound **43** (163 mg, 0,49 mmol, 20% yield). LC-MS $[M+H]^+$: 332 *m*/*z*. ^{1}H-NMR (500 MHz, $CDCl_3$) δ 10.76 (s, 1H), 7.48 (d, *J* = 7.1 Hz, 2H), 7.32–7.22 (m, 8H), 6.97 (t, *J* = 3.0 Hz, 1H), 5.94 (t, *J* = 2.5 Hz, 1H), 5.30 (s, 2H), 5.15 (s, 2H).

General procedure for the preparation of 5-substituted 1H-pyrrolo[3,2-d]pyrimidine-2,4(3H,5H)-dione derivatives **45a–d.**

To the solution of 1,3-dibenzyl-1*H*-pyrrolo[3,2-*d*]pyrimidine-2,4(3*H*,5*H*)-dione (**43**) in NMP, CuBr (2.00 eq), Na_2CO_3 (1.05 eq) and the corresponding aryl bromide (3.30 eq) were added, then the suspension was heated to 170 °C under argon atmosphere for 3 days. The mixture was filtered through celite and washed with THF twice, then purified by reverse phase flash column chromatography. After the isolation of the desired product **44a–d**, it was dissolved in THF (30 mL). 300 m/m% Pd on carbon was added and hydrogenated in autoclave under 5 bar H_2 pressure for 3 days at 170 °C (the pressure at 170 °C was ca. 22 bar). It was cooled to RT, the suspension was filtered through celite and washed with THF. After removal of the solvent, the expected product was obtained.

Synthesis of 5-phenyl-1H-pyrrolo[3,2-d]pyrimidine-2,4(3H,5H)-dione (**45a**).

The reaction was carried out according to the general procedure, starting from 450 mg (1.36 mmol) 1,3-dibenzyl-1*H*-pyrrolo[3,2-*d*]pyrimidine-2,4(3*H*,5*H*)-dione (**43**), 4 mL NMP, 390 mg (2.72 mmol) CuBr, 153 mg (1.44 mmol) Na_2CO_3, 482 μL (4.6 mmol) bromobenzene to give 1,3-dibenzyl-5-phenyl-1*H*-pyrrolo[3,2-*d*]pyrimidine-2,4(3*H*,5*H*)-dione (**44a**, 338 mg, 0.83 mmol, 61% yield). LC-MS $[M+H]^+$: 408 *m*/*z*. ^{1}H-NMR (500 MHz, $CDCl_3$) δ 7.36 (d, *J* = 7.3 Hz, 2H), 7.28–7.14 (m, 9H), 7.14–7.09 (m, 3H), 7.05 (dd, *J* = 8.2, 6.4 Hz, 1H), 6.83 (d, *J* = 3.0 Hz, 1H), 5.87 (d, *J* = 3.0 Hz, 1H), 5.10 (s, 2H), 5.00 (s, 2H). ^{13}C-NMR (500 MHz, $CDCl_3$) δ 154.51, 151.61, 138.39, 137.71, 136.87, 136.15, 131.04, 128.73, 128.67, 128.63, 128.23, 127.81, 127.71, 127.34, 127.20, 125.54, 110.26, 96.22, 48.68, 44.48. The reaction was carried out according to the general procedure, starting from 338 mg (0.83 mmol) 1,3-dibenzyl-5-phenyl-1*H*-pyrrolo[3,2-*d*]pyrimidine-2,4(3*H*,5*H*)-dione (**44a**), 1.01 g Pd on carbon to give 5-phenyl-1*H*-pyrrolo[3,2-*d*]pyrimidine-2,4(3*H*,5*H*)-dione (**45a**, 28.0 mg, 0.125 mmol, 15% yield). LC-MS $[M+H]^+$: 228 m/z. ^{1}H-NMR (500 MHz, DMSO-d_6) δ 10.65 (bs, 2H), 7.46–7.39 (m, 5H), 7.36 (d, *J* = 4.1 Hz, 1H), 6.07 (s, 1H). ^{13}C-NMR (500 MHz, DMSO-d_6) δ 155.01, 151.11, 138.25, 137.29, 131.74, 128.46, 127.00, 125.04, 109.30, 96.51.

Synthesis of 5-(3-methylphenyl)-1H-pyrrolo[3,2-d]pyrimidine-2,4(3H,5H)-dione (**45b**).

The reaction was carried out according to the general procedure, starting from 450 mg (1.36 mmol) 1,3-dibenzyl-1*H*-pyrrolo[3,2-*d*]pyrimidine-2,4(3*H*,5*H*)-dione (**43**), 4 mL NMP, 390 mg (2.72 mmol) CuBr, 153 mg (1.44 mmol) Na_2CO_3, 550 μL (4.60 mmol) 3-bromotoluene to give 1,3-dibenzyl-5-(3-methylphenyl)-1*H*-pyrrolo[3,2-*d*]pyrimidine-2,4(3*H*,5*H*)-dione (**44b**, 388.0 mg, 0.915 mmol, 68% yield). LC-MS $[M+H]^+$: 422 *m*/*z*. ^{1}H-NMR (500 MHz, $CDCl_3$) δ 7.36 (d, *J* = 7.4 Hz, 2H), 7.23–7.15 (m, 6H), 7.13 (t, *J* = 7.2 Hz, 2H), 7.09–7.02 (m, 4H), 6.84 (d, *J* = 2.5 Hz, 1H), 5.88 (d, *J* = 2.5 Hz, 1H), 5.11 (s, 2H), 5.01 (s, 2H), 2.25 (s, 3H). ^{13}C-NMR (500 MHz, $CDCl_3$) δ 154.51, 151.67, 138.64, 138.39, 137.78, 136.77, 136.21, 131.08, 128.75, 128.71, 128.69, 128.43, 128.23,

127.72, 127.36, 127.19, 126.15, 122.89, 110.38, 96.04, 48.69, 44.48, 21.33. The reaction was carried out according to the general procedure, starting from 388 mg (0.92 mmol) 1,3-dibenzyl-5-(3-methylphenyl)-1*H*-pyrrolo[3,2-*d*]pyrimidine-2,4(3*H*,5*H*)-dione (**44b**), 1.16 g Pd on carbon to give 5-(3-methylphenyl)-1*H*-pyrrolo[3,2-*d*]pyrimidine-2,4(3*H*,5*H*)-dione (**45b**, 31.0 mg, 0.128 mmol, 14% yield). LC-MS $[M+H]^+$: 242 m/z. ^{1}H-NMR (300 MHz, DMSO-d_6) δ 10.97 (s, 1H), 10.65 (s, 1H), 7.95 (d, *J* = 6.9 Hz, 1H), 7.61–7.41 (m, 1H), 7.41–7.12 (m, 3H), 6.06 (s, 1H), 2.34 (s, 3H). ^{13}C-NMR (300 MHz, DMSO-d_6) δ 155.06, 151.21, 138.26, 138.00, 137.29, 132.34, 128.31, 127.73, 125.61, 122.27, 109.35, 96.45, 20.87.

Synthesis of 5-(3-methoxyphenyl)-1H-pyrrolo[3,2-d]pyrimidine-2,4(3H,5H)-dione (**45c**).

The reaction was carried out according to the general procedure, starting from 450 mg (1.36 mmol) 1,3-dibenzyl-1*H*-pyrrolo[3,2-*d*]pyrimidine-2,4(3*H*,5*H*)-dione (**43**), 4 mL NMP, 390 mg (2.72 mmol) CuBr, 153 mg (1.44 mmol) Na_2CO_3, 575 µL (4.60 mmol) 3-bromoanisole to give 1,3-dibenzyl-5-(3-methoxyphenyl)-1*H*-pyrrolo[3,2-*d*]pyrimidine-2,4(3*H*,5*H*)-dione (**44c**, 307 mg, 0.72 mmol, 53% yield). LC-MS $[M+H]^+$: 438 *m/z*. ^{1}H-NMR (500 MHz, $CDCl_3$) δ 7.33 (d, *J* = 7.4 Hz, 2H), 7.18–7.04 (m, 8H), 7.01 (t, *J* = 7.3 Hz, 1H), 6.83 (d, *J* = 3.0 Hz, 1H), 6.80–6.75 (m, 2H), 6.71 (dd, *J* = 8.3, 2.0 Hz, 1H), 5.85 (d, *J* = 3.0 Hz, 1H), 5.08 (s, 2H), 4.96 (s, 2H), 3.58 (s, 3H). ^{13}C-NMR (500 MHz, $CDCl_3$) δ 159.36, 154.13, 151.34, 139.15, 137.52, 136.69, 135.95, 130.94, 129.02, 128.46, 128.40, 127.96, 127.44, 127.09, 126.94, 117.46, 113.15, 111.53, 109.94, 96.03, 55.11, 49.04, 44.21. The reaction was carried out according to the general procedure, starting from 307 mg (0.73 mmol) 1,3-dibenzyl-5-(3-methoxyphenyl)-1*H*-pyrrolo[3,2-*d*]pyrimidine-2,4(3*H*,5*H*)-dione (**44c**), 921 mg Pd on carbon to give 5-(3-methoxyphenyl)-1*H*-pyrrolo[3,2-*d*]pyrimidine-2,4(3*H*,5*H*)-dione (**45c**, 40.0 mg, 0.155 mmol, 16% yield). LC-MS $[M+H]^+$: 258 *m/z*. ^{1}H-NMR (500 MHz, DMSO-d_6) δ 10.95 (s, 1H), 10.63 (s, 1H), 7.43 (d, *J* = 2.9 Hz, 1H), 7.33 (t, *J* = 8.1 Hz, 1H), 7.03 (t, *J* = 2.0 Hz, 1H), 7.00 (dd, *J* = 8.0, 1.3 Hz, 1H), 6.93 (dd, *J* = 8.3, 1.9 Hz, 1H), 6.06 (d, *J* = 2.9 Hz, 1H), 3.79 (s, 3H). ^{13}C-NMR (500 MHz, DMSO-d_6) δ 159.14, 155.00, 151.09, 139.31, 137.40, 131.80, 129.20, 117.06, 112.83, 111.05, 109.30, 96.50, 55.31.

Synthesis of 5-(4-methylphenyl)-1H-pyrrolo[3,2-d]pyrimidine-2,4(3H,5H)-dione (**45d).**

The reaction was carried out according to the general procedure, starting from 450 mg (1.36 mmol) 1,3-dibenzyl-1*H*-pyrrolo[3,2-*d*]pyrimidine-2,4(3*H*,5*H*)-dione (**43**), 4 mL NMP, 390 mg (2.72 mmol) CuBr, 153 mg (1.44 mmol) Na_2CO_3, 560 µL (4.60 mmol) 4-bromotoluene to give 1,3-dibenzyl-5-(4-methylphenyl)-1*H*-pyrrolo[3,2-*d*]pyrimidine-2,4(3*H*,5*H*)-dione (**44d,** 380 mg, 0.90 mmol, 66% yield). LC-MS $[M+H]^+$: 422 *m/z*. ^{1}H-NMR (500 MHz, $CDCl_3$) δ 7.36 (d, *J* = 7.3 Hz, 2H), 7.21–7.03 (m, 12H), 6.81 (d, *J* = 3.0 Hz, 1H), 5.85 (d, *J* = 3.0 Hz, 1H), 5.10 (s, 2H), 4.99 (s, 2H), 2.23 (s, 3H). ^{13}C-NMR (500 MHz, $CDCl_3$) δ 154.52, 151.64, 137.78, 137.76, 136.65, 136.20, 135.97, 130.97, 129.24, 128.75, 128.71, 128.20, 127.69, 127.34, 127.18, 125.40, 110.35, 95.93, 48.66, 44.45, 21.06. The reaction was carried out according to the general procedure, starting from 380 mg (0.90 mmol) 1,3-dibenzyl-5-(4-methylphenyl)-1*H*-pyrrolo[3,2-*d*]pyrimidine-2,4(3*H*,5*H*)-dione (**44d**), 1.14 g Pd on carbon to give 5-(4-methylphenyl)-1*H*-pyrrolo[3,2-*d*]pyrimidine-2,4(3*H*,5*H*)-dione (**45d,** 37.0 mg, 0.153 mmol, 17% yield). LC-MS $[M+H]^+$: 242 *m/z*. ^{1}H-NMR (300 MHz, DMSO-d_6) δ 10.98 (s, 1H), 10.63 (s, 1H), 7.35 (d, *J* = 2.7 Hz, 1H), 7.31 (d, *J* = 8.2 Hz, 2H), 7.22 (d, *J* = 8.2 Hz, 2H), 6.04 (d, *J* = 2.6 Hz, 1H), 2.34 (s, 3H). ^{13}C-NMR (300 MHz, DMSO-d_6) δ 155.09, 151.18, 137.12, 136.49, 135.92, 131.69, 128.94, 124.94, 109.36, 96.29, 20.54.

General procedure for the preparation of 8-substituted 3,4-dihydroisoquinoline derivatives **47a–d.**

In a microwave tube, to the solution of 8-chloro-3,4-dihydroisoquinoline hydrochloride (**46**) in a toluene–water mixture (3:1, 4 mL), potassium fluoride (4.00 eq), tetrakis(triphenylphosphine)palladium(0) (0.015 eq), and the corresponding phenylboronic acid (1.2 eq) were added and reacted in a microwave

reactor at 120 °C for 4 h. The phases were separated, the organic layer was dried over $MgSO_4$, filtered and evaporated. The crude product was purified by reverse phase flash column chromatography.

8-Phenyl-3,4-dihydroisoquinoline (**47a**). The reaction was carried out according to the general procedure, starting from 300 mg (1.49 mmol) 8-chloro-3,4-dihydroisoquinolin hydrochloride (**46**), 344 mg (5.93 mmol) potassium fluoride, 25.0 mg (0.022 mmol) tetrakis(triphenylphosphine)palladium(0), 217 mg (1.78 mmol) phenylboronic acid to give 8-phenyl-3,4-dihydroisoquinoline (**47a**, 140 mg, 0.57 mmol, 38% yield). LC-MS $[M+H]^+$: 208 *m/z*. ^{1}H-NMR (500 MHz, $CDCl_3$) δ 8.73 (s, 1H), 7.35 (t, *J* = 7.3 Hz, 2H), 7.29 (t, *J* = 7.3 Hz, 1H), 7.23 (d, *J* = 7.1 Hz, 2H), 7.17 (t, *J* = 7.5 Hz, 1H), 7.07 (d, *J* = 7.6 Hz, 1H), 7.02 (d, *J* = 7.4 Hz, 1H), 3.90–3.85 (m, 2H), 2.91–2.86 (m, 2H).

8-(3-Methylphenyl)-3,4-dihydroisoquinoline (**47b).** The reaction was carried out according to the general procedure, starting from 300 mg (1.49 mmol) 8-chloro-3,4-dihydroisoquinolin hydrochloride (**46**), 344 mg (5.93 mmol) potassium fluoride, 25.0 mg (0.022 mmol) tetrakis(triphenylphosphine)palladium(0), 243 mg (1.78 mmol) *m*-tolylboronic acid to give 8-(3-methylphenyl)-3,4-dihydroisoquinoline (**47b**, 148 mg, 0.67 mmol, 45% yield). LC-MS $[M+H]^+$: 222 *m/z*. ^{1}H-NMR (500 MHz, $CDCl_3$) δ 8.22 (s, 1H), 7.27 (t, *J* = 7.6 Hz, 1H), 7.22 (t, *J* = 7.5 Hz, 1H), 7.16 (d, *J* = 7.0 Hz, 1H), 7.10 (d, *J* = 7.6 Hz, 1H), 7.08–7.03 (m, 3H), 3.66 (t, *J* = 7.4 Hz, 2H), 2.67 (t, *J* = 7.5 Hz, 2H), 2.30 (s, 3H).

8-(3-Methoxyphenyl)-3,4-dihydroisoquinoline (**47c**). The reaction was carried out according to the general procedure, starting from 300 mg (1.49 mmol) 8-chloro-3,4-dihydroisoquinolin hydrochloride (**46**), 344 mg (5.93 mmol) potassium fluoride, 25.0 mg (0.022 mmol) tetrakis(triphenylphosphine)palladium(0), 271 mg (1.78 mmol) (3-methoxyphenyl)boronic acid to give 8-(3-methoxyphenyl)-3,4-dihydroisoquinoline (**47c**, 172 mg, 0.73 mmol, 49% yield). LC-MS $[M+H]^+$: 238 *m/z*. ^{1}H-NMR (500 MHz, $CDCl_3$) δ 8.24 (s, 1H), 7.27 (t, *J* = 7.6 Hz, 1H), 7.24 (t, *J* = 7.9 Hz, 1H), 7.17 (d, *J* = 7.5 Hz, 1H), 7.06 (d, *J* = 7.4 Hz, 1H), 6.86 – 6.81 (m, 2H), 6.80 (d, *J* = 1.5 Hz, 1H), 3.72 (s, 3H), 3.67 (d, *J* = 6.8 Hz, 2H), 2.70–2.64 (m, 2H).

8-(4-Methylphenyl)-3,4-dihydroisoquinoline (**47d**). The reaction was carried out according to the general procedure, starting from 300 mg (1.49 mmol) 8-chloro-3,4-dihydroisoquinolin hydrochloride (**46**), 344 mg (5.93 mmol) potassium fluoride, 25.0 mg (0.022 mmol) tetrakis(triphenylphosphine)palladium(0), 243 mg (1.78 mmol) p-tolylboronic acid to give 8-(4-methylphenyl)-3,4-dihydroisoquinoline (**47d**, 131 mg, 0.61 mmol, 41% yield). LC-MS $[M+H]^+$: 222 *m/z*. ^{1}H-NMR (500 MHz, $CDCl_3$) δ 8.35 (s, 1H), 7.37 (t, *J* = 7.6 Hz, 1H), 7.28–7.23 (m, 5H), 7.14 (d, *J* = 7.4 Hz, 1H), 3.77 (t, *J* = 6.7 Hz, 2H), 2.82–2.73 (m, 2H), 2.41 (s, 3H).

General procedure for the preparation of 8-substituted 1,2,3,4-tetrahydroisoquinoline derivatives **48a–d**, **50a–c.**

To the solution of the corresponding 8-substituted 3,4-dihydroisoquinoline in methanol, $NaBH_4$ (1.1 eq) was added at 0 °C, then the mixture was stirred at RT for 1 h. After the reaction was completed, water was added to the mixture, and washed with DCM twice. The organic phase was dried over $MgSO_4$, filtered and evaporated. The product was used in the next step without further purification.

8-Phenyl-1,2,3,4-tetrahydroisoquinoline (**48a**). The reaction was carried out according to the general procedure, starting from 140 mg (0.68 mmol) 8-phenyl-3,4-dihydroisoquinoline (**47a**), 2.7 mL methanol, 28.0 mg (0.740 mmol) $NaBH_4$ to give 8-phenyl-1,2,3,4-tetrahydroisoquinoline (**48a**, 106 mg, 0.51 mmol, 75% yield). LC-MS $[M+H]^+$: 210 *m/z*. ^{1}H-NMR (500 MHz, $CDCl_3$) δ 7.41 (t, *J* = 7.3 Hz, 2H), 7.36 (t, *J* = 7.2 Hz, 1H), 7.29 (d, *J* = 7.2 Hz, 2H), 7.23 (t, *J* = 7.7 Hz, 1H), 7.13 (d, *J* = 7.6 Hz, 1H), 7.08 (d, *J* = 7.3 Hz, 1H), 3.87 (s, 2H), 3.14 (t, *J* = 6.0 Hz, 2H), 2.91 (t, *J* = 5.9 Hz, 2H), 2.42 (s, 1H).

8-(3-Methylphenyl)-1,2,3,4-tetrahydroisoquinoline (**48b**). The reaction was carried out according to the general procedure, starting from 148 mg (0.67 mmol) 8-(3-methylphenyl)-3,4-dihydroisoquinoline (**47b**), 2.8 mL methanol, 28.0 mg (0.740 mmol) $NaBH_4$ to give 8-(3-methylphenyl)-1,2,3,4-tetrahydroisoquinoline (**48b**, 103 mg, 0.46 mmol, 69% yield). LC-MS $[M+H]^+$: 224 *m/z*. ^{1}H-NMR (500 MHz, $CDCl_3$) δ 7.29 (t, *J* = 7.6 Hz, 1H), 7.22–7.18 (m, 1H), 7.16 (d, *J* = 7.5 Hz, 1H), 7.12–7.04 (m, 4H), 3.87 (s, 2H), 3.14 (t, *J* = 6.0 Hz, 2H), 2.90 (t, *J* = 6.0 Hz, 2H), 2.40 (s, 3H).

8-(3-Methoxyphenyl)-1,2,3,4-tetrahydroisoquinoline (**48c**). The reaction was carried out according to the general procedure, starting from 172 mg (0.73 mmol) 8-(3-methoxyphenyl)-3,4-dihydroisoquinoline (**47c**), 2.7 mL methanol, 30.0 mg (0.793 mmol) $NaBH_4$ to give 8-(3-methoxyphenyl)-1,2,3,4-tetrahydroisoquinoline (**48c**, 123 mg, 0.52 mmol, 71% yield). LC-MS $[M+H]^+$: 240 *m/z*. ^{1}H-NMR (500 MHz, $CDCl_3$) δ 7.30 (t, *J* = 7.9 Hz, 1H), 7.20 (t, *J* = 7.5 Hz, 1H), 7.11 (d, *J* = 7.5 Hz, 1H), 7.06 (d, *J* = 7.4 Hz, 1H), 6.89 (dt, *J* = 5.9, 3.0 Hz, 1H), 6.86 (d, *J* = 7.6 Hz, 1H), 6.83 (s, 1H), 3.87 (s, 2H), 3.81 (s, 3H), 3.13 (t, *J* = 6.0 Hz, 2H), 2.89 (t, *J* = 5.8 Hz, 2H), 2.78 (s, 1H).

8-(4-Methylphenyl)-1,2,3,4-tetrahydroisoquinoline (**48d**). The reaction was carried out according to the general procedure, starting from 131 mg (0.59 mmol) 8-(4-methylphenyl)-3,4-dihydroisoquinoline (**47d**), 2.9 mL methanol, 25.0 mg (0.661 mmol) $NaBH_4$ to give) 8-(4-methylphenyl)-1,2,3,4-tetrahydroisoquinoline (**48d**, 102 mg, 0.46 mmol, 78% yield. LC-MS $[M+H]^+$: 224 *m/z*. ^{1}H-NMR (500 MHz, $CDCl_3$) δ 7.24–7.20 (m, 3H), 7.17 (d, *J* = 8.0 Hz, 2H), 7.11 (d, *J* = 7.6 Hz, 1H), 7.06 (d, *J* = 7.4 Hz, 1H), 3.90 (s, 2H), 3.16 (t, *J* = 6.1 Hz, 2H), 2.93 (t, *J* = 5.9 Hz, 2H), 2.42 (d, *J* = 6.4 Hz, 3H).

4-Methyl-8-piperidin-1-yl-1,2,3,4-tetrahydroisoquinoline (**50a**). The reaction was carried out according to the general procedure, starting from 1.46 g (6.81 mmol) 8-piperidin-1-yl-3,4-dihydroisoquinoline (**49a**), 30 mL methanol, 283 mg (7.49 mmol) $NaBH_4$ to give 4-methyl-8-piperidin-1-yl-1,2,3,4-tetrahydroisoquinoline (**50a**, 1.20 g, 5.59 mmol, 82% yield). LC-MS $[M+H]^+$: 217 *m/z*. ^{1}H-NMR (500 MHz, $CDCl_3$) δ 7.06 (t, *J* = 7.7 Hz, 1H), 6.84 (d, *J* = 7.9 Hz, 1H), 6.75 (d, *J* = 7.6 Hz, 1H), 3.95 (s, 2H), 3.41 (s, 1H), 3.06 (s, 2H), 2.82–2.67 (m, 6H), 1.69–1.60 (m, 4H), 1.53 (s, 2H).

4-Methyl-8-morpholin-4-yl-1,2,3,4-tetrahydroisoquinoline (**50b**). The reaction was carried out according to the general procedure, starting from 1.40 g (6.47 mmol) 8-morpholin-4-yl-3,4-dihydroisoquinoline (**49b**), 28 mL methanol, 270 mg (7.14 mmol) $NaBH_4$ to give 4-methyl-8-morpholin-4-yl-1,2,3,4-tetrahydroisoquinoline (**50b**, 1.13 g, 5.17 mmol, 80% yield). LC-MS $[M+H]^+$: 219 *m/z*. ^{1}H-NMR (500 MHz, $CDCl_3$) δ 7.10 (t, *J* = 7.8 Hz, 1H), 6.86 (d, *J* = 8.0 Hz, 1H), 6.81 (d, *J* = 7.6 Hz, 1H), 3.99 (s, 3H), 3.74 (dd, *J* = 12.3, 7.9 Hz, 4H), 3.11 (t, *J* = 6.2 Hz, 2H), 2.83–2.76 (m, 6H).

4-Methyl-8-pyrrolidin-1-yl-1,2,3,4-tetrahydroisoquinoline (**50c**). The reaction was carried out according to the general procedure, starting from 2.00 g (9.99 mmol) 8-pyrrolidin-1-yl-3,4-dihydroisoquinoline (**49c**), 40 mL methanol, 416 mg (11 mmol) $NaBH_4$ to give 4-methyl-8-pyrrolidin-1-yl-1,2,3,4-tetrahydroisoquinoline (**50c**, 1.76 g, 8.69 mmol, 87% yield). LC-MS $[M+H]^+$: 203 *m/z*. ^{1}H-NMR (500 MHz, $CDCl_3$) δ 7.02 (t, *J* = 7.5 Hz, 1H), 6.72 (d, *J* = 8.0 Hz, 1H), 6.63 (d, *J* = 7.6 Hz, 1H), 3.94 (s, 1H), 3.69 (s, 1H), 3.21–3.09 (m, 6H), 2.93 (dd, *J* = 14.9, 5.0 Hz, 2H), 2.85 (t, *J* = 5.9 Hz, 1H), 2.01–1.90 (m, 4H).

[(Benzyloxy)methyl](triphenyl)phosphonium chloride (**52**). To the solution of [(chloromethoxy)-methyl]benzene (**51**, 11.30 g, 72.2 mmol) in dry THF (100 mL), triphenylphosphine (20.70 g, 78.9 mmol) was added and stirred at 90 °C for 16 h. The white precipitate was filtered and dried in vacuum. [(benzyloxy)methyl](triphenyl)phosphonium chloride (**52**, 23.40 g, 55.9 mmol, 78% yield) was produced. Mp 170-173°C. ^{1}H-NMR (500 MHz, DMSO-d_6) δ 7.93 (td, *J* = 7.2, 3.0 Hz, 3H), 7.84–7.75 (m, 12H), 7.33 (t, *J* = 6.5 Hz, 3H), 7.25–7.21 (m, 2H), 5.76 (d, *J* = 5.0 Hz, 2H), 4.72 (s, 2H).

Ethyl 4-[(E)-2-(benzyloxy)ethenyl]-1H-pyrrole-2-carboxylate (**54**). The suspension of [(benzyloxy)methyl](triphenyl)phosphonium chloride (**52**, 9.00 g, 21.5 mmol) in dry THF (100 mL) was stirred and cooled to −78 °C, then butyllithium solution was added dropwise (7.75 mL, 19.4 mmol), and stirred at −78 °C for 30 min. To this solution, ethyl 4-formyl-1*H*-pyrrole-2-carboxylate (**53**, 0.90 g, 5.38 mmol) dissolved in dry THF (10 mL) was added dropwise and the reaction was warmed to RT and stirred for 16 h. The organic layer was washed with water, dried over $MgSO_4$, filtered and purified by flash chromatography in hexane–ethyl acetate to give ethyl 4-[(*E*)-2-(benzyloxy)ethenyl]-1*H*-pyrrole-2-carboxylate (**54**, 0.80 g, 2.95 mmol, 55% yield)**.** LC-MS $[M+H]^+$: 272 *m*/*z*. ^{1}H-NMR (500 MHz, $CDCl_3$) δ 9.15 (s, 1H), 7.37 (m, 4H), 7.22 (d, *J* = 1.1 Hz, 1H), 7.03 (s, 1H), 6.17 (t, *J* = 5.9 Hz, 1H), 5.27 (d, *J* = 6.5 Hz, 1H), 4.97 (s, 2H), 4.32 (q, *J* = 7.1 Hz, 2H), 1.35 (t, *J* = 7.1 Hz, 3H).

Ethyl 4-(2-hydroxyethyl)-1H-pyrrole-2-carboxylate (**55*).*** To the solution of ethyl 4-[(*E*)-2-(benzyloxy)ethenyl]-1*H*-pyrrole-2-carboxylate (**54**, 0.217 g , 0.8 mmol) in methanol (15 mL), Pd on carbon (0.022 g, 10 m/m%) was added and hydrogenated for 2 days at 110 °C. It was cooled to RT, the suspension was filtered through celite and washed with methanol (3 x 5 mL). After removal of the solvent, ethyl 4-(2-hydroxyethyl)-1*H*-pyrrole-2-carboxylate (**56**, 0.11 g, 0.6 mmol, 75% yield) was obtained. LC-MS $[M+H]^+$: 184 *m*/*z*. ^{1}H-NMR (500 MHz, $CDCl_3$) δ 9.40 (s, 1H), 6.80 (s, 1H), 6.78 (s, 1H), 4.29 (q, *J* = 7.1 Hz, 2H), 3.77 (t, *J* = 6.5 Hz, 2H), 2.71 (t, *J* = 6.5 Hz, 2H), 1.33 (t, *J* = 7.1 Hz, 3H).

Ethyl 4-{2-[(methylsulfonyl)oxy]ethyl}-1H-pyrrole-2-carboxylate (**56**). To the solution of ethyl 4-(2-hydroxyethyl)-1*H*-pyrrole-2-carboxylate (**55**, 1.27 g, 6.89 mmol) in dry THF (140 mL), triethylamine (0.963 mL, 6.89 mmol) and methanesulfonyl chloride (0.535 mL, 6.89 mmol) were added at RT and stirred for 16 h. The crude product was used in the next step without further purification. LC-MS $[M+H]^+$: 262 *m*/*z*.

General procedure for the preparation of ester derivatives **57** *and* **58.**

To the solution of the corresponding linker part **45a**–**d**, **48a**–**d**, **50a**–**c** in acetonitrile, K_2CO_3 and ethyl 4-{2-[(methylsulfonyl)oxy]ethyl}-1*H*-pyrrole-2-carboxylate (**56**) were added and stirred at 100 °C for 16 h. The solvent was evaporated and the crude product was purified by reverse phase flash chromatography.

Ethyl 4-[2-(2,4-dioxo-5-phenyl-1,2,4,5-tetrahydro-3H-pyrrolo[3,2-d]pyrimidin-3-yl)ethyl]-1H-pyrrole-2-carboxylate (**57a**). The reaction was carried out according to the general procedure, starting from 22.0 mg (0.097 mmol) 5-phenyl-1*H*-pyrrolo[3,2-*d*]pyrimidine-2,4(3*H*,5*H*)-dione (**45a**), 27.0 mg (0.194 mmol, 2.00 eq) K_2CO_3, 28 mg (0.10 mmol, 1.10 eq) ethyl 4-{2-[(methylsulfonyl)oxy]ethyl}-1*H*-pyrrole-2-carboxylate (**56**) to give ethyl 4-[2-(2,4-dioxo-5-phenyl-1,2,4,5-tetrahydro-3*H*-pyrrolo[3,2-*d*]pyrimidin-3-yl)ethyl]-1*H*-pyrrole-2-carboxylate (**57a**, 8.0 mg, 0.0204 mmol, 21% yield). LC-MS $[M+H]^+$: 393 *m*/*z*.

Ethyl 4-{2-[5-(3-methylphenyl)-2,4-dioxo-1,2,4,5-tetrahydro-3H-pyrrolo[3,2-d]pyrimidin-3-yl]ethyl}-1H-pyrrole-2-carboxylate (**57b**). The reaction was carried out according to the general procedure, starting from 31.0 mg (0.128 mmol) 5-(3-methylphenyl)-1*H*-pyrrolo[3,2-*d*]pyrimidine-2,4(3*H*,5*H*)-dione (**45b**), 35 mg (0.26 mmol, 2.00 eq) K_2CO_3, 37.0 mg (0.141 mmol, 1.10 eq) ethyl 4-{2-[(methylsulfonyl)oxy]ethyl}-1*H*-pyrrole-2-carboxylate (**56**) to give ethyl 4-{2-[5-(3-methylphenyl)-2,4-dioxo-1,2,4,5-tetrahydro-3*H*-pyrrolo[3,2-*d*]pyrimidin-3-yl]ethyl}-1*H*-pyrrole-2-carboxylate (**57b**, 13.0 mg, 0.032 mmol, 25% yield). LC-MS $[M+H]^+$: 407 *m*/*z*.

Ethyl 4-{2-[5-(3-methoxyphenyl)-2,4-dioxo-1,2,4,5-tetrahydro-3H-pyrrolo[3,2-d]pyrimidin-3-yl]ethyl}-1H-pyrrole-2-carboxylate (**57c**). The reaction was carried out according to the general procedure, starting from 21.0 mg (0.082 mmol) 5-(3-methoxyphenyl)-1*H*-pyrrolo[3,2-*d*]pyrimidine-2,4(3*H*,5*H*)-dione (**45c**), 23.0 mg (0.164

mmol, 2 eq) K_2CO_3, 24 mg (0.09 mmol, 1.1 eq) ethyl 4-{2-[(methylsulfonyl)oxy]ethyl}-1*H*-pyrrole-2-carboxylate (**56**) to give ethyl 4-{2-[5-(3-methoxyphenyl)-2,4-dioxo-1,2,4,5-tetrahydro-3*H*-pyrrolo[3,2-*d*]pyrimidin-3-yl]ethyl}-1*H*-pyrrole-2-carboxylate (**57c**, 7.0 mg, 0.016 mmol, 20% yield). LC-MS $[M+H]^+$: 423 *m/z*.

Ethyl 4-{2-[5-(4-methylphenyl)-2,4-dioxo-1,2,4,5-tetrahydro-3H-pyrrolo[3,2-d]pyrimidin-3-yl]ethyl}-1H-pyrrole-2-carboxylate (**57d**). The reaction was carried out according to the general procedure, starting from 37.0 mg (0.153 mmol) 5-(4-methylphenyl)-1*H*-pyrrolo[3,2-*d*]pyrimidine-2,4(3*H*,5*H*)-dione (**45d**), 42.0 mg (0.306 mmol, 2.00 eq) K_2CO_3, 44 mg (0.17 mmol, 1.10 eq) ethyl 4-{2-[(methylsulfonyl)oxy]ethyl}-1*H*-pyrrole-2-carboxylate (**56**) to give ethyl 4-{2-[5-(4-methylphenyl)-2,4-dioxo-1,2,4,5-tetrahydro-3*H*-pyrrolo[3,2-*d*]pyrimidin-3-yl]ethyl}-1*H*-pyrrole-2-carboxylate (**57d**, 15.0 mg, 0.0367 mmol, 24% yield). LC-MS $[M+H]^+$: 407 *m/z*.

Ethyl 4-[2-(8-phenyl-3,4-dihydroisoquinolin-2(1H)-yl)ethyl]-1H-pyrrole-2-carboxylate (**58a**). The reaction was carried out according to the general procedure, starting from 24.0 mg (0.115 mmol) 8-phenyl-1,2,3,4-tetrahydroisoquinoline (**48a**), 64 mg (0.46 mmol, 4.00 eq) K_2CO_3, 30.0 mg (0.115 mmol, 1.00 eq) ethyl 4-{2-[(methylsulfonyl)oxy]ethyl}-1*H*-pyrrole-2-carboxylate (**56**) to give ethyl 4-[2-(8-phenyl-3,4-dihydro-isoquinolin-2(1*H*)-yl)ethyl]-1*H*-pyrrole-2-carboxylate (**58a**, 18.0 mg, 0.0483 mmol, 42% yield). LC-MS $[M+H]^+$: 375 *m/z*.

Ethyl 4-{2-[8-(3-methylphenyl)-3,4-dihydroisoquinolin-2(1H)-yl]ethyl}-1H-pyrrole-2-carboxylate (**58b**). The reaction was carried out according to the general procedure, starting from 26.0 mg (0.115 mg) 8-(3-methylphenyl)-1,2,3,4-tetrahydroisoquinoline (**48b**), 64 mg (0.46 mmol, 4.00 eq) K_2CO_3, 30.0 mg (0.115 mmol, 1.00 eq) ethyl 4-{2-[(methylsulfonyl)oxy]ethyl}-1*H*-pyrrole-2-carboxylate (**56**) to give ethyl 4-{2-[8-(3-methylphenyl)-3,4-dihydroisoquinolin-2(1*H*)-yl]ethyl}-1*H*-pyrrole-2-carboxylate (**58b**, 8.0 mg, 0.0207 mmol, 18% yield). LC-MS $[M+H]^+$: 389 *m/z*.

Ethyl 4-{2-[8-(3-methoxyphenyl)-3,4-dihydroisoquinolin-2(1H)-yl]ethyl}-1H-pyrrole-2-carboxylate (**58c**). The reaction was carried out according to the general procedure, starting from 28.0 mg (0.115 mmol) 8-(3-methoxyphenyl)-1,2,3,4-tetrahydroisoquinoline (**48c**), 64.0 mg (0.46 mmol, 4.00 eq) K_2CO_3, 30 mg (0.12 mmol, 1.00 eq) ethyl 4-{2-[(methylsulfonyl)oxy]ethyl}-1*H*-pyrrole-2-carboxylate (**56**) to give ethyl 4-{2-[8-(3-methoxyphenyl)-3,4-dihydroisoquinolin-2(1*H*)-yl]ethyl}-1*H*-pyrrole-2-carboxylate (**58c**, 24.0 mg, 0.0598 mmol, 52% yield). LC-MS $[M+H]^+$: 405 *m/z.*

Ethyl 4-{2-[8-(4-methylphenyl)-3,4-dihydroisoquinolin-2(1H)-yl]ethyl}-1H-pyrrole-2-carboxylate (**58d**). The reaction was carried out according to the general procedure, starting from 26.0 mg (0.115 mmol) 8-(4-methylphenyl)-1,2,3,4-tetrahydroisoquinoline (**48d**), 64 mg (0.46 mmol, 4.00 eq) K_2CO_3, 30.0 mg (0.115 mmol, 1.00 eq) ethyl 4-{2-[(methylsulfonyl)oxy]ethyl}-1*H*-pyrrole-2-carboxylate (**56**) to give ethyl 4-{2-[8-(4-methylphenyl)-3,4-dihydroisoquinolin-2(1*H*)-yl]ethyl}-1*H*-pyrrole-2-carboxylate (**58d**, 13.0 mg, 0.0334 mmol, 29% yield). LC-MS $[M+H]^+$: 389 *m/z*.

Ethyl 4-[2-(8-piperidin-1-yl-3,4-dihydroisoquinolin-2(1H)-yl)ethyl]-1H-pyrrole-2-carboxylate (**58e**). The reaction was carried out according to the general procedure, starting from 25.0 mg (0.115 mmol) 4-methyl-8-piperidin-1-yl-1,2,3,4-tetrahydroisoquinoline (**50a**), 64 mg (0.46 mmol, 4.00 eq) K_2CO_3, 30.0 mg (0.115 mmol, 1.00 eq) ethyl 4-{2-[(methylsulfonyl)oxy]ethyl}-1*H*-pyrrole-2-carboxylate (**56**) to give ethyl 4-[2-(8-piperidin-1-yl-3,4-dihydroisoquinolin-2(1*H*)-yl)ethyl]-1*H*-pyrrole-2-carboxylate (**58e**, 16.0 mg, 0.0414 mmol, 36% yield). LC-MS $[M+H]^+$: 382 *m/z.*

Ethyl 4-[2-(8-morpholin-4-yl-3,4-dihydroisoquinolin-2(1H)-yl)ethyl]-1H-pyrrole-2-carboxylate (**58f**). The reaction was carried out according to the general procedure, starting from 25.0 mg (0.115 mmol) 4-methyl-8-morpholin-4-yl-1,2,3,4-tetrahydroisoquinoline (**50b**), 64 mg (0.46 mmol, 4.00 eq) K_2CO_3, 30.0 mg (0.115 mmol, 1.00 eq) ethyl 4-{2-[(methylsulfonyl)oxy]ethyl}-1*H*-pyrrole-2-carboxylate (**56**) to give ethyl 4-[2-(8-morpholin-4-yl-3,4-dihydroisoquinolin-2(1*H*)-yl)ethyl]-1*H*-pyrrole-2-carboxylate (**58f**, 15.0 mg, 0.0391 mmol, 34% yield). LC-MS $[M+H]^+$: 384 *m*/*z*.

Ethyl 4-[2-(8-pyrrolidin-1-yl-3,4-dihydroisoquinolin-2(1*H*)-yl)ethyl]-1*H*-pyrrole-2-carboxylate (**58g**). The reaction was carried out according to the general procedure, starting from 23.0 mg (0.115 mmol) 4-methyl-8-pyrrolidin-1-yl-1,2,3,4-tetrahydroisoquinoline (**50c**), 64 mg (0.46 mmol, 4.00 eq) K_2CO_3, 30.0 mg (0.115 mmol, 1.00 eq) ethyl 4-{2-[(methylsulfonyl)oxy]ethyl}-1*H*-pyrrole-2-carboxylate (**56**) to give ethyl 4-[2-(8-pyrrolidin-1-yl-3,4-dihydroisoquinolin-2(1*H*)-yl)ethyl]-1*H*-pyrrole-2-carboxylate (**58g**, 8.0 mg, 0.0219 mmol, 19% yield). LC-MS $[M+H]^+$: 368 *m*/*z*.

General procedure for the preparation of carboxylic acid derivatives **16–26.**

The solution of the corresponding esters (**57a**–**d**, **58a**–**g**) in dioxane–1 M NaOH (1:1, 4 mL) was stirred for 2 h, then set to pH 7 with 1 M HCl solution and the solvent was removed. The resulting crude product was suspended with ethanol (2 mL) and filtered. After the evaporation of the solvent, the expected product was obtained.

4-[2-(2,4-Dioxo-5-phenyl-1,2,4,5-tetrahydro-3H-pyrrolo[3,2-d]pyrimidin-3-yl)ethyl]-1H-pyrrole-2-carboxylic acid (**16**). The reaction was carried out according to the general procedure, starting from 4.0 mg (0.01 mmol) ethyl 4-[2-(2,4-dioxo-5-phenyl-1,2,4,5-tetrahydro-3*H*-pyrrolo[3,2-*d*]pyrimidin-3-yl)ethyl]-1*H*-pyrrole-2-carboxylate (**57a**) to give 4-[2-(2,4-dioxo-5-phenyl-1,2,4,5-tetrahydro-3*H*-pyrrolo[3,2-*d*]pyrimidin-3-yl)-ethyl]-1*H*-pyrrole-2-carboxylic acid (**16**, 2.9 mg, 0.0079 mmol, 80% yield). ^{1}H-NMR (500 MHz, DMSO-d_6) δ 11.59 (s, 1H), 10.85 (s, 1H), 7.48 (d, *J* = 3.0 Hz, 1H), 7.45–7.42 (m, 2H), 7.37 (td, *J* = 8.5, 3.9 Hz, 1H), 6.90 (s, 1H), 6.73 (s, 1H), 6.44 (d, *J* = 3.1 Hz, 1H), 4.00–3.94 (m, 1H), 2.77–2.73 (m, 1H). ^{13}C-NMR (500 MHz, DMSO-d_6) δ 160.78, 154.80, 150.87, 138.66, 132.30, 127.68, 122.94, 122.23, 121.06, 115.54, 109.91, 97.25, 59.80, 45.23, 25.17. HRMS (ESI$^+$) *m*/*z* $[M+H]^+$ calcd. for $C_{19}H_{16}N_4O_4Na$: 387.1069, found: 387.1068.

4-{2-[5-(3-Methylphenyl)-2,4-dioxo-1,2,4,5-tetrahydro-3H-pyrrolo[3,2-d]pyrimidin-3-yl]ethyl}-1H-pyrrole-2-carboxylic acid (**17**). The reaction was carried out according to the general procedure, starting from 7.0 mg (0.015 mmol) ethyl 4-{2-[5-(3-methylphenyl)-2,4-dioxo-1,2,4,5-tetrahydro-3*H*-pyrrolo[3,2-*d*]pyrimidin-3-yl]ethyl}-1*H*-pyrrole-2-carboxylate (**57b**) to give 4-{2-[5-(3-methylphenyl)-2,4-dioxo-1,2,4,5-tetrahydro-3*H*-pyrrolo[3,2-*d*]pyrimidin-3-yl]ethyl}-1*H*-pyrrole-2-carboxylic acid (**17**, 3.9 mg, 0.010 mmol, 61% yield). ^{1}H-NMR (500 MHz, DMSO-d_6) δ 11.59 (s, 1H), 10.83 (s, 1H), 7.45 (d, *J* = 3.0 Hz, 1H), 7.31 (t, *J* = 7.7 Hz, 1H), 7.24 (s, 1H), 7.21 (d, *J* = 7.9 Hz, 1H), 7.18 (d, *J* = 6.2 Hz, 1H), 6.90 (s, 1H), 6.72 (s, 1H), 6.42 (d, *J* = 3.0 Hz, 1H), 4.00–3.94 (m, 2H), 2.77–2.73 (m, 2H), 2.33 (s, 3H). ^{13}C-NMR (500 MHz, DMSO-d_6) δ 160.76, 154.74, 150.88, 138.85, 138.61, 138.42, 132.26, 128.72, 128.32, 126.18, 122.94, 122.84, 122.23, 122.16, 121.38, 121.06, 115.54, 109.93, 97.11, 59.80, 45.22, 25.17, 21.27. HRMS (ESI$^+$) *m*/*z* $[M+H]^+$ calcd. for $C_{20}H_{18}N_4O_4Na$: 401.1226, found: 401.1220.

4-{2-[5-(3-Methoxyphenyl)-2,4-dioxo-1,2,4,5-tetrahydro-3H-pyrrolo[3,2-d]pyrimidin-3-yl]ethyl}-1H-pyrrole-2-carboxylic acid (**18**). The reaction was carried out according to the general procedure, starting from 3.0 mg (0.0060 mmol) ethyl 4-{2-[5-(3-methoxyphenyl)-2,4-dioxo-1,2,4,5-tetrahydro-3*H*-pyrrolo[3,2-*d*]pyrimidin-3-yl]ethyl}-1*H*-pyrrole-2-carboxylate (**57c**) to give 4-{2-[5-(3-methoxyphenyl)-2,4-dioxo-1,2,4,5-tetrahydro-3*H*-pyrrolo[3,2-*d*]pyrimidin-3-yl]ethyl}-1*H*-pyrrole-2-carboxylic acid (**18**, 1.5 mg, 0.0038 mmol, 63% yield). ^{1}H-NMR (500 MHz, DMSO-d_6) δ 11.59 (s, 1H), 10.83 (s, 1H), 7.50 (d, *J* = 3.1 Hz, 1H), 7.33 (t, *J* = 8.1 Hz, 1H), 7.03

(t, *J* = 2.2 Hz, 1H), 7.00 (dd, *J* = 7.9, 1.2 Hz, 1H), 6.93 (dd, *J* = 8.3, 2.4 Hz, 1H), 6.90 (s, 1H), 6.73 (s, 1H), 6.43 (d, *J* = 3.1 Hz, 1H), 4.01–3.95 (m, 2H), 3.77 (s, 3H), 2.78–2.71 (m, 2H). ^{13}C-NMR (500 MHz, DMSO-*d*$_6$) δ 162.81, 160.77, 159.59, 154.75, 150.85, 139.70, 139.00, 129.67, 122.94, 122.23, 121.05, 117.72, 115.55, 113.50, 111.66, 109.91, 59.80, 55.82, 44.18, 25.61, 25.17, 14.86. HRMS (ESI$^+$) *m*/*z* [M+H]$^+$ calcd. for $C_{20}H_{19}N_4O_5$: 395.1355, found: 395.1357.

4-{2-[5-(4-Methylphenyl)-2,4-dioxo-1,2,4,5-tetrahydro-3*H*-pyrrolo[3,2-*d*]pyrimidin-3-yl]ethyl}-1*H*-pyrrole-2-carboxylic acid (**19**). The reaction was carried out according to the general procedure, starting from 7.0 mg (0.018 mmol) ethyl 4-{2-[5-(4-methylphenyl)-2,4-dioxo-1,2,4,5-tetrahydro-3*H*-pyrrolo[3,2-*d*]pyrimidin-3-yl]ethyl}-1*H*-pyrrole-2-carboxylate (**57d**) to give 4-{2-[5-(4-methylphenyl)-2,4-dioxo-1,2,4,5-tetrahydro-3*H*-pyrrolo[3,2-*d*]pyrimidin-3-yl]ethyl}-1*H*-pyrrole-2-carboxylic acid (**19**, 3.8 mg, 0.010 mmol, 63% yield). ^{1}H-NMR (500 MHz, DMSO-*d*$_6$) δ 11.59 (s, 1H), 10.88–10.74 (m, 1H), 7.42 (d, *J* = 3.0 Hz, 1H), 7.30 (d, *J* = 8.3 Hz, 2H), 7.23 (d, *J* = 8.3 Hz, 2H), 6.90 (s, 1H), 6.72 (t, *J* = 2.0 Hz, 1H), 6.41 (d, *J* = 3.1 Hz, 1H), 4.00–3.94 (m, 2H), 2.77–2.72 (m, 2H), 2.34 (s, 3H). ^{13}C-NMR (500 MHz, DMSO-*d*$_6$) δ 160.76, 154.80, 150.87, 138.70, 137.13, 136.30, 132.19, 122.92, 122.23, 122.16, 121.38, 121.07, 115.53, 109.96, 96.98, 59.80, 45.21, 25.17, 20.99. HRMS (ESI$^+$) *m*/*z* [M+H]$^+$ calcd. for $C_{20}H_{18}N_4O_4Na$: 401.1226, found: 401.1228.

4-[2-(8-Phenyl-3,4-dihydroisoquinolin-2(1*H*)-yl)ethyl]-1*H*-pyrrole-2-carboxylic acid (**20**). The reaction was carried out according to the general procedure, starting from 9.0 mg (0.024 mmol) ethyl 4-[2-(8-phenyl-3,4-dihydroisoquinolin-2(1*H*)-yl)ethyl]-1*H*-pyrrole-2-carboxylate (**58a**) to give 4-[2-(8-phenyl-3,4-dihydro-isoquinolin-2(1*H*)-yl)ethyl]-1*H*-pyrrole-2-carboxylic acid (**20**, 5.9 mg, 0.017 mmol, 71% yield). ^{1}H-NMR (500 MHz, DMSO-*d*$_6$) δ 11.50 (s, 1H), 10.65 (s, 1H), 7.47 (dd, *J* = 11.4, 4.4 Hz, 2H), 7.37–7.32 (m, 1H), 7.28 (dt, *J* = 10.2, 3.7 Hz, 3H), 7.24 (s, 1H), 7.15–7.12 (m, 2H), 6.80 (d, *J* = 2.4 Hz, 1H), 6.60 (t, *J* = 2.0 Hz, 1H), 4.32 (dd, *J* = 15.4, 8.0 Hz, 1H), 4.14 (d, *J* = 15.0 Hz, 1H), 3.77–3.69 (m, 1H), 3.40–3.23 (m, 4H), 2.92–2.84 (m, 1H), 2.79–2.71 (m, 1H). ^{13}C-NMR (500 MHz, DMSO-*d*$_6$) δ 162.12, 133.10, 128.54, 124.00, 123.44, 122.26, 119.92, 118.32, 114.87, 56.46, 53.81, 49.65, 49.01, 26.28, 25.64, 21.75. HRMS (ESI$^+$) *m*/*z* [M+H]$^+$ calcd. for $C_{22}H_{23}N_2O_2$: 347.1760, found: 347.1758.

4-{2-[8-(3-Methylphenyl)-3,4-dihydroisoquinolin-2(1H)-yl]ethyl}-1H-pyrrole-2-carboxylic acid (**21**). The reaction was carried out according to the general procedure, starting from 4.0 mg (0.01 mmol) ethyl 4-{2-[8-(3-methylphenyl)-3,4-dihydroisoquinolin-2(1*H*)-yl]ethyl}-1*H*-pyrrole-2-carboxylate (**58b**) to give 4-{2-[8-(3-methylphenyl)-3,4-dihydroisoquinolin-2(1*H*)-yl]ethyl}-1*H*-pyrrole-2-carboxylic acid (**21**, 3.0 mg, 0.0083 mmol, 76% yield). ^{1}H-NMR (500 MHz, DMSO-*d*$_6$) δ 11.50 (s, 1H), 10.75 (s, 1H), 7.37–7.32 (m, 3H), 7.28–7.20 (m, 3H), 7.15–7.08 (m, 3H), 7.06 (d, *J* = 7.7 Hz, 1H), 6.80 (s, 1H), 6.60 (t, *J* = 2.0 Hz, 1H), 4.29 (dd, *J* = 15.7, 7.8 Hz, 1H), 4.13 (d, *J* = 15.2 Hz, 1H), 3.72 (d, *J* = 7.0 Hz, 1H), 2.92–2.84 (m, 1H), 2.79–2.72 (m, 1H), 2.34 (d, *J* = 4.8 Hz, 3H). ^{13}C-NMR (500 MHz, DMSO-*d*$_6$) δ 161.60, 140.20, 138.89, 137.83, 132.04, 129.24, 128.40, 128.30, 128.03, 127.76, 127.55, 125.63, 122.87, 121.71, 119.01, 114.32, 66.96, 55.48, 51.37, 47.78, 21.00. HRMS (ESI$^+$) *m*/*z* [M+H]$^+$ calcd. for $C_{23}H_{25}N_2O_2$: 361.1916, found: 361.1917.

4-{2-[8-(3-Methoxyphenyl)-3,4-dihydroisoquinolin-2(1H)-yl]ethyl}-1H-pyrrole-2-carboxylic acid (**22**). The reaction was carried out according to the general procedure, starting from 12.0 mg (0.03 mmol) ethyl 4-{2-[8-(3-methoxyphenyl)-3,4-dihydroisoquinolin-2(1*H*)-yl]ethyl}-1*H*-pyrrole-2-carboxylate (**58c**) to give 4-{2-[8-(3-methoxyphenyl)-3,4-dihydroisoquinolin-2(1*H*)-yl]ethyl}-1*H*-pyrrole-2-carboxylic acid (**22**, 9.4 mg, 0.025 mmol, 83% yield). ^{1}H-NMR (500 MHz, DMSO-*d*$_6$) δ 11.50 (s, 1H), 11.24 (s, 1H), 7.35–7.32 (m, 2H), 7.25 (s, 1H), 7.13 (d, *J* = 6.8 Hz, 1H), 6.98–6.94 (m, 1H), 6.84 (t, *J* = 4.2 Hz, 2H), 6.81 (s, 1H), 6.60–6.59 (m, 1H), 4.31 (dd, *J* = 15.5, 7.7 Hz, 1H), 4.16 (t, *J* = 12.3 Hz, 1H), 3.77 (s, 3H), 3.36–3.28 (m, 4H), 2.95–2.87 (m, 1H), 2.83–2.75 (m, 1H). ^{13}C-NMR (500 MHz, DMSO-*d*$_6$) δ 171.90, 162.07, 159.69, 140.83, 140.42, 132.65, 130.14, 128.41, 128.33,

127.95, 126.25, 123.33, 122.16, 121.30, 119.59, 114.80, 114.74, 113.74, 55.91, 55.63, 51.58, 49.00, 48.06, 34.42, 25.63, 21.50. HRMS (ESI+) *m/z* [M+H]+ calcd. for $C_{23}H_{25}N_2O_3$: 377.1865, found: 377.1867.

4-{2-[8-(4-Methylphenyl)-3,4-dihydroisoquinolin-2(1H)-yl]ethyl}-1H-pyrrole-2-carboxylic acid (**23**). The reaction was carried out according to the general procedure, starting from 6.0 mg (0.016 mmol) ethyl 4-{2-[8-(4-methylphenyl)-3,4-dihydroisoquinolin-2(1*H*)-yl]ethyl}-1*H*-pyrrole-2-carboxylate (**58d**) to give 4-{2-[8-(4-methylphenyl)-3,4-dihydroisoquinolin-2(1*H*)-yl]ethyl}-1*H*-pyrrole-2-carboxylic acid (**23**, 4.9 mg, 0.014 mmol, 85% yield). ^{1}H-NMR (500 MHz, DMSO-*d*6) δ 11.50 (s, 1H), 11.08 (s, 1H), 7.35 (d, *J* = 7.6 Hz, 1H), 7.27–7.23 (m, 3H), 7.16 (d, *J* = 8.0 Hz, 2H), 7.10 (d, *J* = 7.2 Hz, 1H), 6.81 (s, 1H), 6.60 (s, 1H), 4.28 (dd, *J* = 15.4, 7.8 Hz, 1H), 4.12 (d, *J* = 14.9 Hz, 1H), 3.70 (d, *J* = 8.6 Hz, 1H), 2.93–2.85 (m, 1H), 2.82–2.74 (m, 1H), 2.35 (s, 3H). HRMS (ESI+) *m/z* [M+H]+ calcd. for $C_{23}H_{25}N_2O_2$: 361.1916, found: 361.1915.

4-[2-(8-Piperidin-1-yl-3,4-dihydroisoquinolin-2(1H)-yl)ethyl]-1H-pyrrole-2-carboxylic acid (**24**). The reaction was carried out according to the general procedure, starting from 8.0 mg (0.021 mmol) ethyl 4-[2-(8-piperidin-1-yl-3,4-dihydroisoquinolin-2(1*H*)-yl)ethyl]-1*H*-pyrrole-2-carboxylate (**58e**) to give 4-[2-(8-piperidin-1-yl-3,4-dihydroisoquinolin-2(1*H*)-yl)ethyl]-1*H*-pyrrole-2-carboxylic acid (**24**, 5.8 mg, 0.0164 mmol, 78% yield). ^{1}H-NMR (500 MHz, DMSO-*d*6) δ 11.57 (s, 1H), 11.13 (s, 1H), 7.24 (t, *J* = 7.8 Hz, 1H), 7.03 (s, 1H), 6.95 (d, *J* = 7.7 Hz, 1H), 6.90 (d, *J* = 2.0 Hz, 1H), 6.69 (d, *J* = 1.9 Hz, 1H), 4.44 (s, 1H), 3.73–3.65 (m, 2H), 3.44 (dd, *J* = 13.4, 8.4 Hz, 2H), 3.37 (dd, *J* = 11.6, 5.6 Hz, 2H), 3.00 (dd, *J* = 18.6, 11.2 Hz, 4H), 2.79 (s, 2H), 2.69 (s, 2H), 1.50 (s, 3H). HRMS (ESI+) *m/z* [M+H]+ calcd. for $C_{21}H_{28}N_3O_2$: 354.2182, found: 354.2187.

4-[2-(8-Morpholin-4-yl-3,4-dihydroisoquinolin-2(1H)-yl)ethyl]-1H-pyrrole-2-carboxylic acid (**25**). The reaction was carried out according to the general procedure, starting from 7.0 mg (0.019 mmol) ethyl 4-[2-(8-morpholin-4-yl-3,4-dihydroisoquinolin-2(1*H*)-yl)ethyl]-1*H*-pyrrole-2-carboxylate (**58f**) to give 4-[2-(8-morpholin-4-yl-3,4-dihydroisoquinolin-2(1*H*)-yl)ethyl]-1*H*-pyrrole-2-carboxylic acid (**25**, 5.5 mg, 0.0155 mmol, 81% yield). ^{1}H-NMR (500 MHz, DMSO-*d*6) δ 11.58 (s, 1H), 11.42 (s, 1H), 7.25 (t, *J* = 7.8 Hz, 1H), 7.04 (d, *J* = 7.9 Hz, 1H), 6.97 (d, *J* = 7.6 Hz, 1H), 6.90 (s, 1H), 6.69 (s, 1H), 4.46 (d, *J* = 14.8 Hz, 1H), 4.17 (dd, *J* = 15.0, 7.6 Hz, 2H), 3.74–3.63 (m, 6H), 3.28 (d, *J* = 9.1 Hz, 2H), 3.00 (dd, *J* = 17.5, 9.9 Hz, 3H), 2.86–2.79 (m, 2H), 2.71–2.65 (m, 2H). ^{13}C-NMR (500 MHz, DMSO-*d*6) δ 162.09, 150.34, 133.26, 128.54, 124.50, 124.26, 123.41, 122.23, 120.04, 118.37, 114.86, 66.88, 56.42, 52.67, 49.54, 48.99, 48.53, 25.61, 21.62. HRMS (ESI+) *m/z* [M+H]+ calcd. for $C_{20}H_{26}N_3O_3$: 356.1974, found: 356.1977.

4-[2-(8-Pyrrolidin-1-yl-3,4-dihydroisoquinolin-2(1H)-yl)ethyl]-1H-pyrrole-2-carboxylic acid (**26**). The reaction was carried out according to the general procedure, starting from 4.0 mg (0.011 mmol) ethyl 4-[2-(8-pyrrolidin-1-yl-3,4-dihydroisoquinolin-2(1*H*)-yl)ethyl]-1*H*-pyrrole-2-carboxylate (**58g**) to give 4-[2-(8-pyrrolidin-1-yl-3,4-dihydroisoquinolin-2(1*H*)-yl)ethyl]-1*H*-pyrrole-2-carboxylic acid (**26**, 1.9 mg, 0,0056 mmol, 52% yield). ^{1}H-NMR (500 MHz, DMSO-*d*6) δ 11.07 (s, 1H), 6.98 (t, *J* = 7.8 Hz, 1H), 6.71 (d, *J* = 7.9 Hz, 1H), 6.69 (s, 1H), 6.64 (d, *J* = 7.3 Hz, 1H), 6.48 (s, 1H), 3.41 (s, 2H), 3.00 (t, *J* = 6.3 Hz, 4H), 2.79 (t, *J* = 5.8 Hz, 2H), 2.68–2.59 (m, 6H), 2.35–2.33 (m, 2H). HRMS (ESI+) *m/z* [M+H]+ calcd. for $C_{20}H_{26}N_3O_2$: 340.2025, found: 340.2023.

References

1. Farnaby, W.; Fieldhouse, C.; Hazel, K.; Kerr, C.; Kinsella, N.; Livermore, D.; Merchant, K.; Miller, D.. Pyridazinone compounds and their use as DAAO inhibitors; Int. Publ. Num.: WO2013027000.

2. Yuasa, Y.*; Tsuruta, H.; Yuasa, Y. Facile Synthesis of β-Keto Esters from Methyl Acetoacetate and Acid Chloride: The Barium Oxide/Methanol System1. **1998**, 1996–1998.

3. Alvarez-Builla, J.; Vaquero, J.J.; Garcia Navio, J.L.; Cabello, J.F.; Sunkel, C.; Fau de Casa-Juana, M.; Dorrego, F.; Santos, L. 1,5-Bis- (N-benzyl-N,N-diethylammoninm) diethylether, dichloride (BBDE C1). A novel bis-ammonium salt as phase transfer catalyst. *Tetrahedron* **1990**, *46*, 967–978.

4. Tsukayama, M.; Kikuchi, M.; Kawamura, Y. Regioselective Prenylation of Phenols by Palladium Catalyst: Syntheses of Prenylphenols and Chromans. *Heterocycles* **1994**, *38*, 1487.

5. Thongsornkleeb, C.; Rabten, W.; Bunrit, A.; Tummatorn, J.; Ruchirawat, S. Facile access to 2,5-disubstituted-4-chloromethyl-3-iodofuran derivatives via ICl-mediated cyclization of 1-alkyl-2-alkynylallylic alcohols. *Tetrahedron Lett.* **2012**, *53*, 6615–6619.

6. Terry-Lorenzo, R.T.T.; Chun, L.E.E.; Brown, S.P.P.; Heffernan, M.L.L.R.; Fang, Q.K.K.; Orsini, M.A.A.; Pollegioni, L.; Hardy, L.W.W.; Spear, K.L.L.; Large, T.H.H. Novel human D-amino acid oxidase inhibitors stabilize an active-site lid-open conformation. *Biosci. Rep.* **2014**, *34*, 487–499.

7. Bourke, D.G.; Burns, C.J.; Cuzzupe, A.N.; Feutrill, J.T.; Kling, M.R.; Nero, T.L.. N-containing heterocyclic compounds; Int. Publ. Num.: WO2009062258.

8. Hargitai, C.; Nagy, T.; Halász, J.; Koványi-Lax, G.; Németh, G.; Simig, G.; Volk, B. Synthesis and further transformations of 8-chloro-3,4-dihydroisoquinoline. *Tetrahedron* **2018**, *74*, 7009−7017.
